# Effects of enhanced adsorption haemofiltration versus haemoadsorption in severe, refractory septic shock with high levels of endotoxemia: the ENDoX bicentric, randomized, controlled trial

**DOI:** 10.1186/s13613-023-01224-8

**Published:** 2023-12-14

**Authors:** Pedro David Wendel-Garcia, Barbara Eberle, Eva-Maria Kleinert, Matthias Peter Hilty, Stephan Blumenthal, Katharina Spanaus, Patricia Fodor, Marco Maggiorini

**Affiliations:** 1 Institute of Intensive Care Medicine, University Hospital Zurich, Rämistrasse 100, 8091 Zurich, Switzerland; 2 Institute of Anaesthesiology, Triemli Hospital, Zurich, Switzerland; 3 Institute of Clinical Chemistry, University Hospital Zurich, Zurich, Switzerland; 4 Institute of Intensive Care Medicine, Triemli Hospital, Zurich, Switzerland

**Keywords:** Haemoperfusion, Haemoadsorption, oXiris, Polymyxin B, Endotoxin, Cytokine Removal, Septic Shock

## Abstract

**Background:**

Endotoxin adsorption is a promising but controversial therapy in severe, refractory septic shock and conflicting results exist on the effective capacity of available devices to reduce circulating endotoxin and inflammatory cytokine levels.

**Methods:**

Multiarm, randomized, controlled trial in two Swiss intensive care units, with a 1:1:1 randomization of patients suffering severe, refractory septic shock with high levels of endotoxemia, defined as an endotoxin activity ≥ 0.6, a vasopressor dependency index ≥ 3, volume resuscitation of at least 30 ml/kg/24 h and at least single organ failure, to a haemoadsorption (Toraymyxin), an enhanced adsorption haemofiltration (oXiris) or a control intervention. Primary endpoint was the difference in endotoxin activity at 72-h post-intervention to baseline. In addition, inflammatory cytokine, vasopressor dependency index and SOFA-Score dynamics over the initial 72 h were assessed inter alia.

**Results:**

In the 30, out of 437 screened, randomized patients (10 Standard of care, 10 oXiris, 10 Toraymyxin), endotoxin reduction at 72-h post-intervention-start did not differ among interventions (Standard of Care: 12 [1–42]%, oXiris: 21 [10–51]%, Toraymyxin: 23 [10–36]%, *p* = 0.82). Furthermore, no difference between groups could be observed neither for reduction of inflammatory cytokine levels (*p* = 0.58), nor for vasopressor weaning (*p* = 0.95) or reversal of organ injury (*p* = 0.22).

**Conclusions:**

In a highly endotoxemic, severe, refractory septic shock population neither the Toraymyxin adsorber nor the oXiris membrane could show a reduction in circulating endotoxin or cytokine levels over standard of care.

*Trial registration* ClinicalTrials.gov. NCT01948778. Registered August 30, 2013. https://clinicaltrials.gov/study/NCT01948778

**Supplementary Information:**

The online version contains supplementary material available at 10.1186/s13613-023-01224-8.

## Introduction

Septic shock remains one of the main mortality causes in critically ill patients and to date no targeted intervention has been able to sustainably change the course of disease [1, 2]. Lipopolysaccharide (LPS) or endotoxin, a constituent part of the gram-negative bacterial wall, is one of the best-known and most studied inductors of inflammatory immune response, which deranging into a cytokine storm, induces the syndrome called septic shock.

In the therapeutic targeting of septic shock great interest and hope has lain in the purification of blood—extracting and eliminating noxious substances, such as LPS—by means of extracorporeal devices, so called haemoadsorption. In this setting, and from as early as 1994, the Polymyxin B adsorber column (Toraymyxin), a polystyrene fiber immobilized antibiotic with the capacity to adsorb and inhibit LPS, has been postulated to reduce the amount of circulating LPS, thus eliminating the immunologic trigger sustaining the cytokine cascade, potentially resulting in reversal of septic shock [3]. More recently, the oXiris filter, a three-layered device coupling LPS with cytokine adsorption, haemofiltration and an antithrombotic priming [4], has been shown to efficiently adsorb LPS and cytokines in-vitro [5] and reverse hemodynamic instability in in-vivo animal septic shock models [6].

Multiple smaller-scale trials have investigated the Toraymyxin and oXiris effect in septic populations, with heterogeneous results ranging from clear survival benefits to futility [7–14]. Nevertheless, results in large, heterogeneous trials have been disappointing to date, with only post-hoc subgroup analyses being able to show an outcome benefit from the therapy [15–17]. Notably, to date only two trials did enrich the studied population by solely including patients with septic shock and high levels of endotoxemia [14, 16]. This although the Endotoxin Activity Assay (EAA) enables a fast bedside assessment of the absolute circulating endotoxin burden [18, 19].

In light of the contradictory evidence on endotoxin adsorption, the question arises if haemoadsorption and enhanced adsorption haemofiltration devices have the capacity to achieve their primarily postulated effect, that is to reduce circulating endotoxin levels in severe, refractory septic shock with high levels of endotoxemia in comparison with standard of care (SOC). The main hypothesis investigated in this trial was, therefore, that treatment with either the oXiris or Toraymyxin device would lead to decreased endotoxin blood levels compared to control.

## Methods

### Trial design and population

The ENDoX-Trial was a bicentric, multiarm, randomized, controlled trial (RCT) conducted in two tertiary Swiss Intensive Care Units. The study was approved by the Swiss regional cantonal ethical commission (EK-ZH 2012-0458, ClinicalTrials.gov NCT01948778, registered 30.08.2013) and complies with the Declaration of Helsinki, the Guidelines on Good Clinical Practice (GCP-Directive) issued by the European Medicines Agency, as well as the Swiss law and Swiss regulatory authority requirements.

Patients with a diagnosed septic shock [20] were eligible for inclusion into the study in the 24 h ensuing diagnosis, if they suffered: (I) a severe, refractory septic shock defined as: (a) volume resuscitation of at least 30 ml/kg in the last 24 h, (b) a Vasopressor Dependency Index [9] (VDI), a surrogate for the cumulative vasoactive requirement of a patient to maintain a specific mean arterial pressure (Additional file 1: Annex S1), above or equal to 3 and (c) either metabolic acidosis, a neurologic dysfunction with a Glasgow Coma Scale (GCS) of 14 or below, acute kidney injury as defined by a RIFLE [21] stage of Injury or Failure or acute liver insufficiency defined by transaminases of more than two times the upper limit of normality; (II) had an EAA value of 0.6 or above and (III) were above 18 years of age.

Exclusion criteria were defined as: (1) contraindication on ethical grounds, (2) child bearing or breastfeeding women, (3) neutropenia defined as ≤ 500 cells/µl, (4) Immunosuppressive Therapy or Steroid doses above or equal to 30 mg/d Prednisone equivalent, (6) use of Vasopressin, (7) organ transplant in the last 12 months, (8) terminal patients, (9) active bleeding defined as (a) thrombocytes after substitution < 30 × 10^6^/l or (b) international normalized ratio > 4 or (c) more than 2 erythrocyte concentrates in a 6-h interval, (10) known allergy to Polymyxin B or anticoagulants, (11) need for extracorporeal membrane oxygenation, (12) participation in another intervention study, (13) no given consent.

### Study intervention

Study participants were randomized equally (1:1:1) to a Toraymyxin intervention, an oXiris intervention or SOC as soon as they were included. Refer to Additional file 2 for the full trial protocol.

The Toraymyxin arm consisted of two haemoadsorption interventions with the Toraymyxin (Toray Industries Inc., Tokyo) separated by a 24-h interval. The adsorber was run on the EstorFlow (Estor S.P.A., Pero) hardware over an interval of 2 h with a blood flow of 150 ml/min. The oXiris arm on the other hand involved a continuous haemodiafiltration with the oXiris (Baxter International Inc., Deerfield) for 48 h at a blood flow of 150 ml/min. The oXiris membrane was mounted on a Prismaflex System (Baxter International Inc., Deerfield) and changed every 24 h. See Additional file 1: Annex S1 for specifications on haemodiafiltration settings and anticoagulation strategy.

There were no further mandatory stipulations to the intervention arms; treatment was left solely to the treating physician’s discretion following the precepts of the Surviving Sepsis Campaign [22], including the use of low-dose corticosteroids. The third arm, serving as non-intervention comparative, was the SOC for septic shock; there were no limitations to the use of hemofiltration when indicated. Hemofiltration, when required in the SOC or Toraymyxin arm, was performed through either the Prismaflex-System with the ST150-membrane (Baxter International Inc., Deerfield) or the MultiFiltrate-System with the AV1000s-membrane (Fresenius Medical Care AG, Bad Homburg). Blood flow settings ranged between 100 and 150 ml/min and were adjusted following weight-corrected, evidence-based, institutional guidelines; anticoagulation was performed with heparin.

Use of Vasopressin was banned for all arms for the duration of 72 h after intervention start. In case of filter clotting in the intervention arms an additional filter was installed to reach the pre-stipulated filter times.

### Primary outcome

The primary endpoint of this RCT was the reduction in EAA at 72-h post-intervention start, assessed between study arms. The EAA shows a near-linear correlation with the absolute LPS concentration in blood, thus enabling a longitudinal assessment of absolute endotoxin levels over time [18, 19], see Additional file 1: Annex S2.

### Secondary outcomes

Secondary endpoints were defined as the compared intervention effects on (I) High-Mobility-Group-Protein B1 (HMGB-1), (II) Interleukin-6, (III) Procalcitonin, (IV) C-reactive protein (CRP) and (V) lactate levels as well as (VI) amount of vasopressors/VDI/Inotropic Score [9], (VII) PaO_2_/FiO_2_ ratio, (VIII) extended hemodynamic parameters and (IX) SOFA-Score over the course of the intervention period. Further endpoints were (X) length of stay in ICU as well as (XI) number and severity of adverse events*.*

### Blood samples and data collection

Blood samples were drawn for every patient during the screening period to measure Endotoxin Activity by means of the Endotoxin Activity Assay (Spectral Medical Inc., Toronto); the highest measured EAA during these 24 h was registered. Every 24 h from inclusion, for a period of 72 h, additional venous and arterial blood samples were drawn and all predefined clinical and laboratory parameters were documented.

### Consent

In this emergency intervention study, a surrogate written informed consent process involving the next of kin, in case of the patient’s inability to consent, as well as an independent physician agreement was prerequisite for the enrolment. Written informed consent from the patient himself was sought as soon as possible and in case of the patient dying or being unable to consent, a definite written informed consent from the next of kin or legal representative was collected.

### Safety

All adverse and serious adverse events occurring during the study interventions as well as in the time frame of 28-day post-study inclusion were collected, noted and reported in compliance with the Swiss stipulations on the performance of clinical studies and medical device studies.

### Statistical analysis

In light of the limited evidence on endotoxin adsorption kinetics, a small cohort of 90 septic shock patients (EAA ≥ 0.6) under SOC was observed, presenting a mean reduction in EAA of 10% ± 16% over 72 h [23]. Expecting a threefold higher reduction in EAA in the filter groups than in the SOC group [6], with a power of 80% and a type-I error probability of 0.05, the targeted sample size per group was calculated to be 10 patients.

Statistical analysis was performed through a fully scripted data management pathway using the R environment for statistical computing version 4.0.1 [24]. Timepoints chosen for statistical inference were the intervention start point defined as hour 0 and timepoints at 24 h, 48 h and 72 h after intervention start. Differences between timepoints and intervention groups were tested using linear mixed effects model analysis. As independent variable fixed effects timepoint and randomization arm were entered into the model. As random effects, intercepts for subjects as well as per-subject random slopes for the effect on dependent variables were employed. *p* values were calculated using Satterthwaite’s method of approximation. Comparisons of population characteristics were performed using the analysis of variance or Kruskal–Wallis test, as appropriate, and the Chi-squared test for categorical variables. A two-sided *p* < 0.05 was considered statistically significant. Values are given as median with interquartile ranges or proportions and percentages as appropriate.

## Results

### Population characteristics

**Fig. 1:**
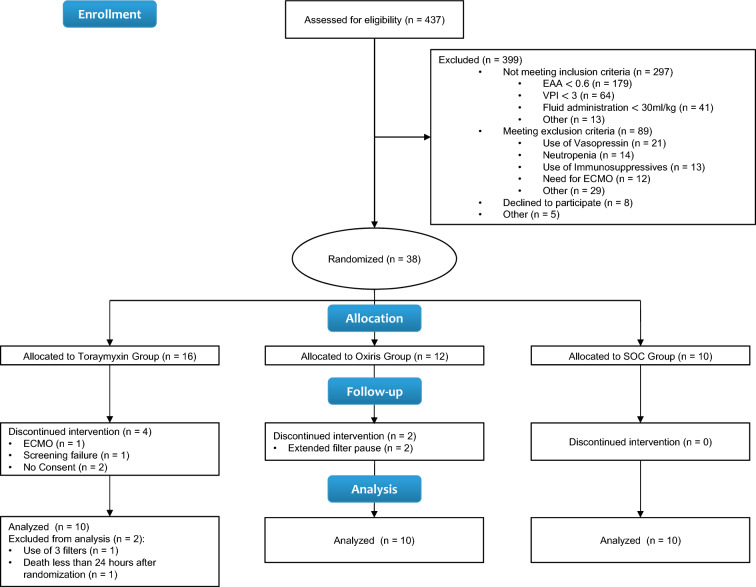
Screening, randomization, allocation and analysis flow of the trial

Figure 1 presents the randomization algorithm, patient allocation, screening failures and study exclusions. Of the 437 patients diagnosed with septic shock and screened for the study between the 31st of August 2013 and the 23rd of May 2019, 38 fulfilled all inclusion and exclusion criteria and were randomized to one of the three study arms, 30 were included into the final analysis.

The included population was characterized by a median age of 71 [66–74] years and had a septic shock of mostly abdominal (15, 50%) and pulmonary (9, 30%) origin, with an equal distribution of gram negative (14, 47%) and positive (13, 43%) bacteria. Patients required a norepinephrine dose of 0.47 [0.29–0.57] μg/kg/min, presented lactate levels of 3 [1.7–4.1] mmol/l as well as EAA levels of 0.71 [0.65–0.78], and had a SOFA score of 13 [11–15], respectively, SAPS II score of 68 [64–72], at study inclusion. All baseline and population characteristics, excepting CRP levels and thrombocyte count were homogenously distributed between groups (Table 1 and Additional file 1: Table S1). Median time between screening and intervention start was 6 [4–8] h for all three groups.

**Table 1:** Baseline and population characteristics

	All patients	Standard of care	oXiris	Toraymyxin
*N*	30	10	10	10
Age, years	71 [66–74]	72 [69–76]	71 [60–72]	71 [61–74]
Sex, male	22 (73%)	7 (70%)	9 (90%)	6 (60%)
BMI, kg m^−2^	27 [25–30]	27 [25–31]	26 [25–29]	27 [25–31]
Time between Screening and Intervention Start, hours	6 [4–8]	5 [4–8]	6 [4–9]	5 [3–9]
SOFA Score	13 [11–15]	14 [11–15]	12 [10–14]	14 [12–15]
SAPS II Score	68 [64–72]	68 [65–70]	68 [60–72]	72 [66–75]
Mean Arterial Pressure, mmHg	64 [50–76]	62 [52–73]	66 [52–81]	62 [50–74]
Norepinephrine dose, µg/kg/min	0.47 [0.29–0.57]	0.48 [0.31–0.57]	0.42 [0.30–0.49]	0.40 [0.26–0.61]
Vasopressor Dependency Index	6.7 [4.1–8.5]	7.1 [4.6–8.5]	6.2 [4.2–8.29]	5.5 [3.4–9.3]
PaO_2_/FiO_2_ Ratio, mmHg	224 [141–304]	205 [110–357]	217 [150–252]	228 [193–323]
Endotoxin Activity	0.71 [0.65–0.78]	0.72 [0.67–0.78]	0.68 [0.66–0.79]	0.72 [0.62–0.74]
Leucocytes, 10^6^/l	13[7–19]	18 [13–22]	9 [6–14]	12 [7–19]
C-Reactive Protein^b^, mg/l	262 [193–338]	366 [242–460]	182 [122–208]	296 [235–334]
Interleukin-6, ng/l	2493 [945–6578]	2226 [945–6126]	3736 [1199–26553]	2722 [509–6513]
Procalcitonin, µg/l	11.7 [3.9–23.9]	10.2 [2.8–14.4]	12.8 [8.8–21.0]	8.3 [3.3–107.6]
Lactate, mmol/l	3.0 [1.7–4.1]	2.7 [2.3–3.4]	3.0 [2.0–4.3]	3.6 [1.4–5.0]
Renal Replacement Therapy^a^	26 (87%)	7 (70%)	10 (100%)	9 (90%)
Mechanical Ventilation	26 (87%)	8 (80%)	8 (80%)	10 (100%)
Positive Blood Cultures	14 (47%)	4 (40%)	4 (40%)	6 (60%)
Length of ICU Stay, days	9 [6–18]	7 [6–11]	9 [6–13]	9 [7–25]
Length of Hospital Stay, days	20 [8–41]	19 [6–40]	14 [11–24]	30 [11–50]
Survival by day 28	22 (73%)	8 (80%)	7 (70%)	7 (70%)

^a^In all cases continuous veno-venous hemodiafiltration was initiated in the first 24 h after study inclusion. ^b^*p* value < 0.05. *ICU* Intensive Care Unit, *SOFA* Sequential Organ Failure Assessment Score, *SAPS II* Simplified Acute Physiology Score II, *PaO*_*2*_ partial pressure of arterial oxygen, *FiO*_*2*_ Fraction of Inspired Oxygen. All values given as median [IQR] or number count (proportion), as appropriate*.* For further baseline characteristics, see Additional file 1: Table S1

### Primary outcome

**Fig. 2:**
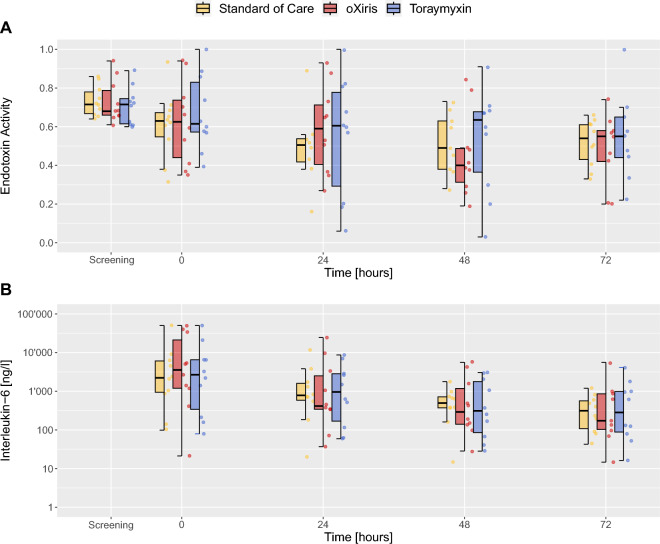
Endotoxin activity and Interleukin-6 kinetics. Boxplots with median and interquartile range, whiskers extend from the difference of the first quartile and 1.5 times the interquartile range, to the sum of the third quartile and 1.5 times the interquartile range. Dots represent the individual measurements. Depicted in yellow, is the Standard of Care arm, in red, with individual, is the oXiris arm and in blue, is the Toraymyxin arm. The *x*-axis portrays the timepoint of screening, the moment of initiation of the individual therapies (defined as 0 h) and 24 h, 48 h and 72 h onwards. Panel-array A presents the Endotoxin Activity and panel-array B the Interleukin-6 levels for the different intervention arms (Standard of Care, oXiris and Toraymyxin, respectively)

In Table 2A and Fig. 2A, the progression and reduction of EAA over time is shown.

**Table 2:** Reduction in endotoxin activity, Interleukin-6, procalcitonin and HMGB-1 over time

Intervention arm	Filter intervention				*p*—over groups	*p*—over timepoints
	0 h	24 h	48 h	72 h		
A. Reduction in endotoxin activity [%]					*0.82*	< *0.01*
Standard of care	0	16 [10–28]	2 [− 18–21]	12 [1–42]		
oXiris	0	2 [− 18–21]	32 [− 8–53]	21 [10–51]		
Toraymyxin	0	12 [1–42]	15 [− 4–34]	23 [10–36]		
B. Reduction in Interleukin-6 [%]					*0.58*	< *0.001*
Standard of care	0	57 [43–74]	77 [61–85]	80 [59–91]		
oXiris	0	75 [28–86]	83 [71–96]	96 [91–98]		
Toraymyxin	0	66 [31–82]	81 [65–95]	85 [48–95]		
C. Reduction in Procalcitonin [%]					*0.16*	< *0.01*
Standard of care	0	− 9 [− 38–29]	34 [11–50]	60 [42–68]		
oXiris	0	31 [− 5–53]	60 [34–75]	71 [27–81]		
Toraymyxin	0	11 [− 55–34]	25 [− 56–62]	18 [− 15–71]		
D. Reduction in High-Mobility-Group-Protein B1 [%]					*0.68*	*0.46*
Standard of care	0	12 [− 7–21]	30 [− 15–52]	32 [− 4–50]		
oXiris	0	23 [− 2–44]	24 [3–31]	37 [14–62]		
Toraymyxin	0	15 [− 18–24]	24 [− 25–27]	21 [− 47–29]		

All parameters (median [IQR]) are given at intervention start (0 h), 24 h, 48 h and 72 h. Absolute *p* values are given over groups and over time. For absolute inflammatory marker levels as well as additional laboratory markers measured, refer to Additional file 1: Table S2

The italic values reflect *p*-values, and are intended to differentiate statistics from numeric values

The median reduction in EAA after 72 h was 12 [1–42]% in the SOC, 21 [10–51]% in the oXiris and 23 [10–36] % in the Toraymyxin arm, as such no difference between arms was patent (*p* = 0.82). Regarding the reduction of EAA after 48 h, timepoint at which all study filter interventions had finished, albeit the oXiris filter presented a higher median EAA reduction (32 [− 8–53]%) in comparison with the Toraymyxin (15 [− 4–34]%) and SOC (2 [− 18–21]%) group, statistical significance between groups was not given (*p* = 0.9).

### Secondary outcomes

Table 2B, C and Fig. 2B show the impact of the different interventions on selected markers of inflammation over time; no difference in HMGB-1, Interleukin-6 and Procalcitonin clearance could be observed.

**Fig. 3:**
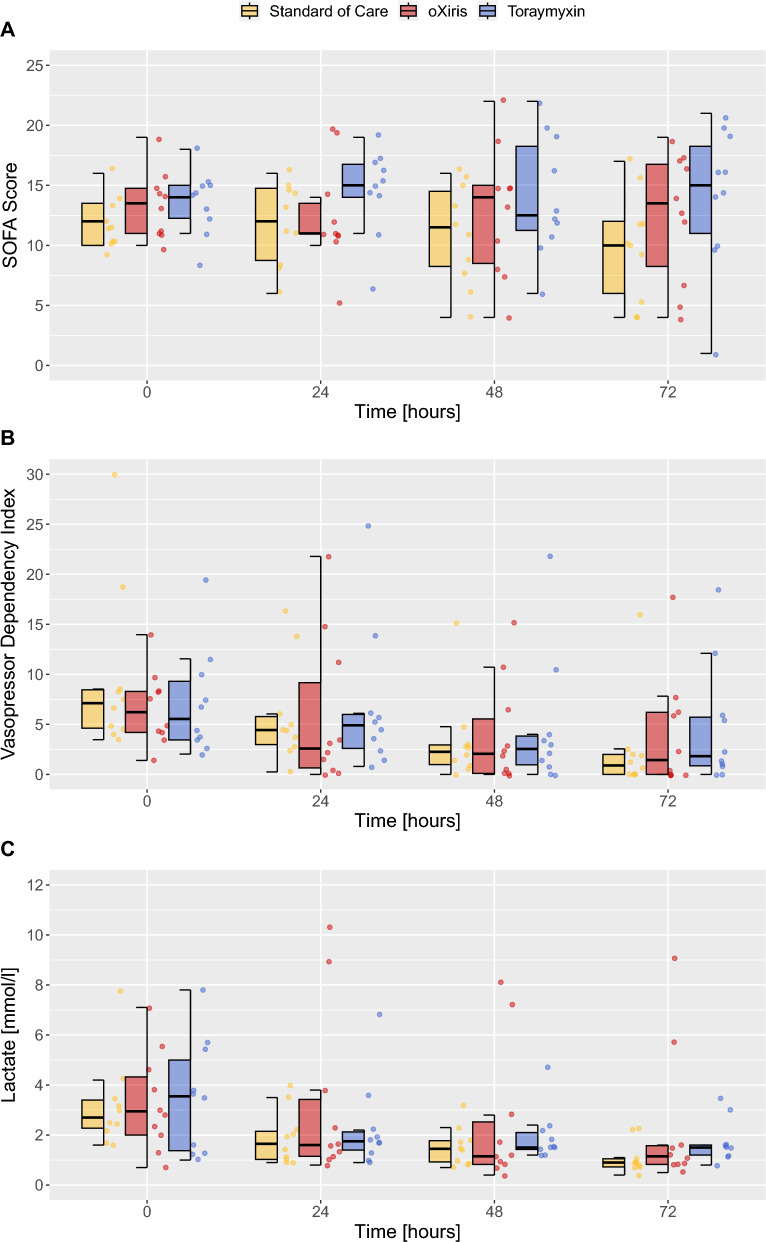
SOFA score, vasopressor dependency index and lactate kinetics. Boxplots with median and interquartile range, whiskers extend from the difference of the first quartile and 1.5 times the interquartile range, to the sum of the third quartile and 1.5 times the interquartile range. Dots represent the individual measurements. Depicted in yellow, is the Standard of Care arm, in red, with individual, is the oXiris arm and in blue, is the Toraymyxin arm. The *x*-axis portrays the moment of initiation of the individual therapies (defined as 0 h) and 24 h, 48 h and 72 h onwards. Panel-array A presents the SOFA Score, panel-array B the Vasopressor Dependency Index and panel-array C lactate levels for the different intervention arms (Standard of Care, oXiris and Toraymyxin, respectively)

There were no differences between groups regarding organ function recovery as assessed by the SOFA Score or Lactate levels (Fig. 3, Additional file 1: Table S3). Furthermore, no difference could be seen between intervention arms regarding VDI, Inotropic Score, norepinephrine dosage nor mean arterial pressure after 72 h (Additional file 1: Table S3, S4). The oXiris and Toraymyxin arm presented lower Thrombocyte counts after 72 h compared to SOC (Additional file 1: Table S2).

### Safety

In total, 14 Adverse Events were reported, 36% (5) in the oXiris Arm, 43% (7) in the Toraymyxin Arm and 14% (2) in the Standard of Care Arm. The most frequently reported serious event was death (Toraymyxin: 3, oXiris: 3, SOC: 2) (Additional file 1: Figure S1); furthermore, three patients in the Toraymyxin arm suffered worsening of their septic shock and one suffered from hemodynamic instability, none of these serious events could be linked to the filters being studied **(**Additional file 1: Table S5). Two adverse events were reported due to clotting of the filter in the oXiris arm, they did not have any deleterious effects for the patients.

## Discussion

This multiarm randomized, controlled study elucidates the adsorption kinetics of two promising adsorbers for Endotoxin adsorption, the Toraymyxin and oXiris filter, in a severe, refractory septic shock population with elevated EAA above or equal to 0.6. No difference in endotoxin levels, as measured by the EAA, could be observed neither in the group being treated with the oXiris filter, nor the Toraymyxin filter when compared to the control population after 72 h. In addition, no effects on cytokine adsorption, vasopressor weaning or reversal of organ injury were patent among intervention arms.

Lipopolysaccharide and inflammatory cytokines have become an increasingly popular target for the treatment of septic shock over the last decades. Haemoadsorption has taken over a prominent role in the search to interfere with the mechanics sustaining the cytokine storm responsible for the septic shock syndrome, after drug targeting of endotoxins failed to show any beneficial effect on the outcome of septic shock patients [25–27].

The recently published studies by Malard et al. and Romanschin et al. showed the excellent adsorption capacity of the oXiris and Toraymyxin filter for endotoxin in in-vitro models [5, 28]. Nevertheless, in the aforementioned studies, plasma and blood were primed with pre-specified amounts of endotoxin and inflammatory cytokines and thus the results only present the hypothetic adsorption capacity of the filters for a fixed endotoxin or cytokine burden. Bassi et al. and Rimmelé et al. showed that the Toraymyxin and a precursor version of the oXiris filter, respectively, managed to clear endotoxin more effectively in septic animal models than controls [6, 29]. However, therapy in the animal models was initiated shortly after “septic induction” with a fixed bacterial load, possibly not leaving enough time for the animal to decompensate to the same extent as in patients with severe, septic shock admitted to an ICU several hours to days after initiation of bacteremia [30]. Furthermore, the reduced distribution volume in the 30–35 kg weighting pigs should also be taken into consideration, and could explain an improved clearance of endotoxins and cytokines.

Similarly, previous experiences with the oXiris filter described significant reductions of endotoxin levels in patients treated with the oXiris filter [12, 14]. An observation that this study supports, but probably pertains more to the natural clearance of endotoxins than to an effect of the filters themselves, when considering the EAA levels in the SOC arm. Furthermore, the fact that the Toraymyxin filter did not show any significant reduction in endotoxin burden in this trial, is not surprising, when regarding the changes in EAA within and between treatment arms in the EUPHRATES trial, the largest Polymyxin B haemoadsorption randomized control trial to date [16].

Why the studied adsorbers are not capable of reducing endotoxin levels in a more efficient fashion than SOC, can be traced back to multiple hypothesis. First of all the adsorption capacity of the filters may lay significantly under the effective systemic clearance of endotoxin. In addition, it can also be that high levels of endotoxins lead to a fast filter saturation without substantial reduction in absolute endotoxemia [17]. Second, a continuous diffusion of endotoxins from highly endotoxin saturated, peripheral compartments towards the generated endotoxin gradient in the central compartment could negate the effect of haemoadsorption, especially affecting patients treated with the Toraymyxin because of the short filter running times [31]. This can also be true for patients in which a secondary bacterial translocation from the gut is responsible for endotoxemia [31], or patients with extremely elevated endotoxin levels (EAA > 0.9) [17]. Thus, longer lasting haemoadsorption therapies coupled with frequent membrane changes could be an effective strategy [32, 33]. Furthermore, the possibility exists that haemoadsorption devices are not capable of adsorbing the type of endotoxins measured by the EAA [34]. Finally, and in line with the theory stated by Matsumoto et al., the EAA may not be indicated to measure absolute values of endotoxin in blood, and possibly only reflect the state of activation the neutrophils find themselves in [35]. A state that is more dependent on the overall inflammatory dysregulation than a causality of endotoxemic burden.

In contrast to previous studies that could show a reduction in inflammatory cytokines by the oXiris filter, the present study could not reproduce any of these effects [12, 14]. Similar unfavourable results have been recently observed for other filters enabling indiscriminate adsorption of cytokines in septic shock [36, 37]. Notably, neither the amount of vasopressors, nor the severity of organ dysfunction could be significantly reduced by either of the device therapies, thus questioning a significant interference of the cytokine storm by endotoxin adsorption. This favours the hypothesis that the hit generated by endotoxins, may only be important during the induction phase of septic shock and once the ensuing pro-inflammatory or immunoparalitical cascade has initiated, endotoxins do not possess a sustaining role [38, 39]. On the other hand, it could also be that the effect haemoadsorption of endotoxins exerts on the systemic immune-derangement can possibly only be noticed after a larger period of time than the one chosen in this study.

### Limitations

The presented study has several limitations. First and foremost this study was designed to capture the kinetics of endotoxin and selected cytokines under haemadsoption and to compare them to a non-intervention group, it was not powered for statistical inference of any therapeutic benefit. Second, and in line with the previous limitation, the number of patients planed per group could have been too small to infer an effect on EAA or cytokine levels. Third, no blinding between groups was undergone for the treating physician team, thus potentially patients could have been treated differently depending on the randomized arm. Fourth, EAA and Interleukin-6 were used as surrogates to the absolute endotoxin load and pro-inflammatory cytokine levels in blood, respectively. Fifth, we included patients with EAA levels above 0.9, which are potential non-responsive to haemadsoption therapies due to their extreme endotoxemic burden [17]. Sixth, only the direct effect of cytokine reduction in response to endotoxin removal can be assessed as some patients received renal replacement with the ST150 membrane, which is a AN69-based membrane absorbing a large spectrum of cytokines without endotoxin absorbing capacity. Finally, the observation period was fixed on a 72-h interval post randomization, precluding evaluation of any effects appearing at a later stage, which could very well be the more relevant phase of endotoxin and cytokine clearance, given the severity of the patients.

## Conclusion

The present bicentric, multiarm, randomized controlled trial comparing the efficiency the oXiris membrane and the Toraymyxin adsorber to a non-intervention control group in patients with severe, refractory septic shock and high endotoxemic burden, could not show that an intervention with neither device could decrease endotoxin activity or inflammatory cytokine levels, as compared to standard of care.

### Supplementary Information

**Additional file 1: Annex S1.** Vasopressor Dependency Index, oXiris Haemodialysis Settings and Anticoagulation Strategy. **Annex S2.** Rationale for the use of the Endotoxin Activity Assay. **Table S1.** Extended Baseline and Population Characteristics. **Table S2.** Extended Inflammation Biomarkers. **Table S3.** SOFA Score, Lactate levels and Vasopressor Index over time. **Table S4.** Extended Hemodynamic Parameters and PaO2/ FiO2 Ratio. **Figure S1.** Mortality at 28 Days in All patients stratified by Intervention Arm. **Table S5.** Reported AEs & SAEs.

**Additional file 2. **Full Study Protocol.

## Data Availability

The corresponding author may provide specified analyses or fully de-identified parts of the data set upon reasonable request.
