# Supplementary material for: Effects of enhanced adsorption haemofiltration versus haemoadsorption in severe, refractory septic shock with high levels of endotoxemia: the ENDoX bicentric, randomized, controlled trial
Source: Ann Intensive Care. 2023 Dec 14;13:127. doi: 10.1186/s13613-023-01224-8 (PMC10721780; doi:10.1186/s13613-023-01224-8)
Supplement: Supplementary file 1 — Additional file 1: Annex S1. Vasopressor Dependency Index, oXiris Haemodialysis Settings and Anticoagulation Strategy. Annex S2. Rationale for the use of the Endotoxin Activity Assay. Table S1. Extended Baseline and Population Characteristics. Table S2. Extended Inflammation Biomarkers. Table S3. SOFA Score, Lactate levels and Vasopressor Index over time. Table S4. Extended Hemodynamic Parameters and PaO2/ FiO2 Ratio. Figure S1. Mortality at 28 Days in All patients stratified by Intervention Arm. Table S5. Reported AEs & SAEs. [file 13613_2023_1224_MOESM1_ESM.pdf]

Supplemental Online Material

Wendel-Garcia, PD., Eberle B., Kleinert EM., Hilty, MP., Blumenthal S., Katharina Spanaus, Fodor P., Maggiorini M.

Effects of enhanced adsorption haemofiltration versus haemoadsorption in severe, refractory septic shock with high levels of endotoxemia: The ENDoX bicentric, randomized, controlled trial

- **Annex S1:** Vasopressor Dependency Index, oXiris Haemodialysis Settings and Anticoagulation Strategy
- **Annex S2:** Rationale for the use of the Endotoxin Activity Assay
- **Table S1:** Extended Baseline and Population Characteristics
- **Table S2:** Extended Inflammation Biomarkers
- **Table S3:** SOFA Score, Lactate levels and Vasopressor Index over time
- **Table S4:** Extended Hemodynamic Parameters and PaO_2/ FiO_2 Ratio
- **Figure S1:** Mortality at 28 Days in All patients stratified by Intervention Arm
- **Table S5:** Reported AEs & SAEs

Annex S1: Vasopressor Dependency Index, oXiris Haemodialysis Settings and Anticoagulation Strategy

Vasopressor Dependency Index

$$\frac{Dobutamine\ Dose + Dopamine\ Dose + \ (Norepinephrine\ Dose \times 100) + (Epinephrine\ Dose \times 100)}{Mean\ Arterial\ Pressure}$$

All Doses are expressed as μg/kg/min.

oXiris - Haemodialysis Settings

- Modality: CVVHDF: 20% Diffusion + 80% Convection
- Initial haemodyalisis dose: 35 ml/h/kg
- Reinfusion: 50% Predilution + 50% Postdilution
- Blood flow: 150ml/min
- Filtration fraction: 10 - 15%
- Replacement and Dialysate Fluid: Haemosol BO® (Bicarbonate 32mmol/l)

Anticoagulation Strategy

- Standard prophylactic anticoagulation with unfractioned Heparin
- Initial Heparin Dose: 8 IU/kg/h (Over filter for oXiris and Toraymyxin)
- Monitoring: First anti-FXa to be measured 1 hour after Heparin start, subsequently every 6 hours
- Target:
  - Post Filter anti-FXa: 0.3-0.6 IU/ml
  - Systemic anti-FXa: <0.3 IU/ml

Annex S2: Rationale for the use of the Endotoxin Activity Assay

To date only semi-quantitative testing methods for the absolute lipopolysaccharide (LPS) burden in blood exits. The most prominent one is the so called, limulus amebocyte lysate (LAL) assay and is based on a lysate of blood cells from the horseshoe crab which can be employed to detect endotoxin levels either via chromogenic testing, gel clotting or turbidimetry. The Endotoxin Activity Assay (EAA) on the other hand is a bedside available method, which employs the oxidative burst of activated neutrophils in response to complement coated LPS-IgM immune complexes, to determine the absolute endotoxin load in blood.

Romaschin et al.[1, 2] have shown that the dose-response curve of the EAA is comparable if not superior to the LAL-Assay in the determination of absolute LPS concentrations in blood, *see Figure 1*. Moreover, the logarithmical part of the dose-response curve above 0.9 EAA does not negate the near-linear correlation between LPS and EAA between 0.2 and 0.9 EAA, *see Figure 2*. This linear response have been shown to be independent of the employed LPS preparation, Figure 3. As such the use of EAA is, given the available methods to measure absolute endotoxin levels to date, equivalent to the gold standard in the quantitative assessment of LPS levels in blood.

1. Romaschin AD, Harris DM, Ribeiro MB, et al. A rapid assay of endotoxin in whole blood using autologous neutrophil dependent chemiluminescence. Journal of Immunological Methods. 1998;212(2):169-185.

2. Romaschin AD, Klein DJ, Marshall JC. Bench-to-bedside review: Clinical experience with the endotoxin activity assay. Critical Care. 2012;16(6):248.

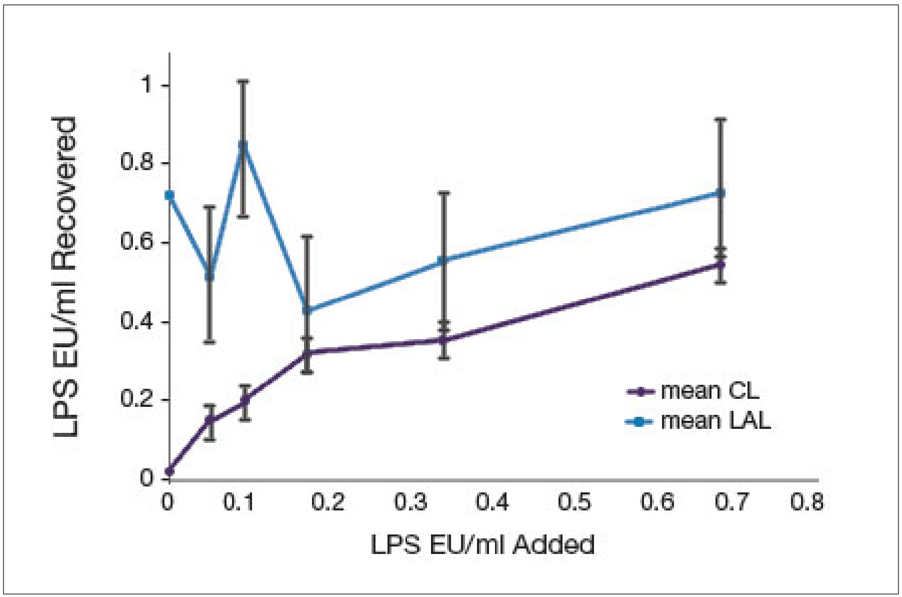

Figure 1: Recovery of LPS added in vitro to whole blood using the acid extraction chromogenic LAL assay panel, versus the chemoluminescence (CL) based EAA. E. Coli 055:B5 LPS was added to whole blood at concentrations of 0, 50, 100, 200, 400 and 800 pg/ml and incubated for 30 min with gentle agitation at 25°C. This blood, taken from eight separate healthy donors, was then split for assay by the LAL and CL procedures. Each data point represents a mean (n=8) ± SEM. *Extracted from Romaschin, A. D., Harris, D. M., Ribeiro, M. B., Paice, J., Foster, D. M., Walker, P. M., & Marshall, J. C. (1998). A rapid assay of endotoxin in whole blood using autologous neutrophil dependent chemi-luminescence. Journal of immunological methods, 212(2), 169-185.*

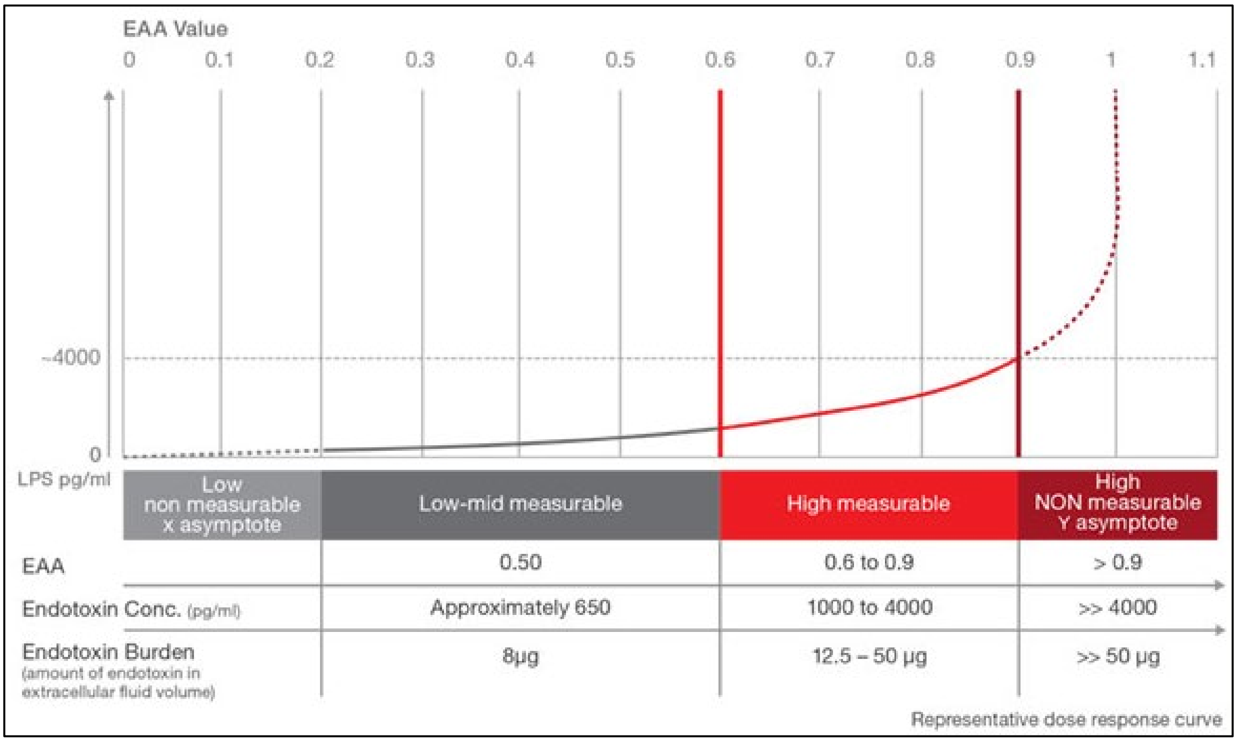

Figure 2: Dose-response curve of EAA versus LPS levels in blood and stratification of endotoxemic burden into low (EAA <0.40), intermediate (EAA 0.40 – 0.59) and high (EAA >0.6). *Extracted from https://spectraldx.com/eaa-for-clinicans/*

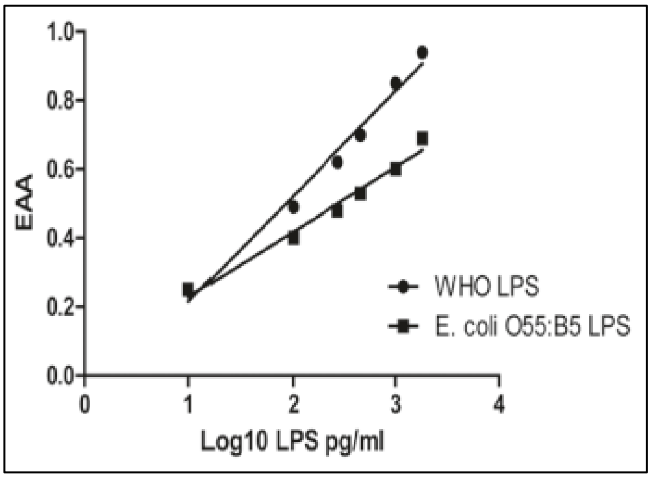

Figure 3: Linearized dose response with EAA plotted against the log(10) LPS concentration for two different LPS preparations. *Extracted from Romaschin, A. D., Klein, D. J., & Marshall, J. C. (2012). Bench-to-bedside review: clinical experience with the endotoxin activity assay. Critical Care, 16(6), 248.*

Table S1: Extended Baseline and Population Characteristics

	All Patients	Standard of Care	Oxiris	Toraymyxin
**N**	30	10	10	10
**Heart Rate, 1/min**	97 [84 - 114]	88 [81 - 96]	110 [90 - 120]	102 [94 - 123]
**Central Venous Pressure, mmHg**	12 [9 - 14]	12 [10 - 12]	11 [8 - 13]	12 [9 - 16]
**Central Venous Oxygen Saturation, %**	70 [68 - 75]	71 [69 - 73]	70 [68 - 74]	72 [67 - 76]
**Cardiac Output, l/min**	7.8 [6 - 9]	7.5 [5.7 - 8.1]	8.8 [5.6 - 9.9]	8.2 6.9 - 9.2]
**Extravascular Lung Water, ml/kg**	10 [8 - 12]	8 [8 - 12]	11 [10 - 13]	9 [7 - 10]
**pH**	7.31 [7.27 - 7.41]	7.33 [7.31 - 7.41]	7.32 [7.26 - 7.41]	7.29 [7.27 - 7.38]
**PaO_2, kPa**	11 [10 - 13]	11 [9 - 13]	12 [10 - 14]	11 [10 - 12]
**FiO_2, %**	42 [30 - 60]	38 [29 - 77]	46 [40 - 60]	37 [31 - 44]
**PEEP, cmH2O**	8 [7 - 12]	8 [8 - 12]	8 [7 - 10]	8 [6 - 12]
**Temperature [°C]**	36.9 [36.4 - 37.8]	36.9 [36.6 - 37.4]	37.0 [36.5 - 37.6]	36.9 [36.3 - 37.8]
**Thrombocytes†, 10^6/l**	160 [102 - 251]	290 [210 - 362]	104 [62 - 144]	167 [138 - 233]
**HMGB-1 [ng/ml]**	2.92 [2.24 – 3.96]	3.21 [2.09 – 4.03]	3.19 [2.58 – 7.73]	2.67 [2.14 – 3.19]
**Fluid Balance, ml/kg/24h**	57 [35 - 75]	66 [37 - 76]	61 [35 - 83]	53 [43 - 67]
**Proven Microorganism**				
Gram negative	14 (47%)	5 (50%)	4 (40%)	5 (50%)
Gram positive	13 (43%)	4 (40%)	4 (40%)	5 (50%)
**Focus of Infection**				
Abdominal	15 (50%)	4 (40 %)	5 (50%)	6 (60 %)
Pulmonary	9 (30%)	4 (40 %)	3 (30%)	2 (20 %)
Skin and Bones	4 (13%)	2 (20 %)	1 (10%)	1 (10 %)
Urogenital	1 (3%)	0 (0%)	1 (10%)	0 (0%)
Catheter-retalted	1 (3%)	0 (0%)	0 (0%)	1 (10%)
**Surgical Source Control Required**	21 (70%)	7 (70%)	8 (80%)	6 (60%)
**Serum Creatinine, µmol/l**	248 [130 - 316]	311 [211 - 407]	152 [128 - 273]	270 [210 - 286]
**KDIGO Score**				
0	3 (10%)	0 (0%)	2 (20%)	1 (10%)
1	1 (3%)	0 (0%)	1 (10%)	0 (0%)
2	4 (13%)	2 (20%)	2 (20%)	0 (0%)
3	22 (73%)	8 (80%)	5 (50%)	9 (90%)
**CRRT with ST150 Membrane**	7 (23%)	2 (20%)	0 (0%)	5 (50%)
**CRRT with AV1000 Membrane**	9 (30%)	7 (70%)	0 (0%)	2 (20%)
**Intensive Care Unit Survival**	23 (77%)	8 (80%)	8 (80%)	7 (70%)

CRRT: Continuous Renal Replacement Therapy | KDIGO: Kidney Disease Improving Global Outcomes | PaO_2: partial pressure of arterial oxygen | FiO_2: Fraction of Inspired Oxygen | PEEP: Positive End-Expiratory Pressure | † p-value < 0.05 | All values given as median [IQR] or number count (proportion), as appropriate.

Table S2: Extended Inflammation Biomarkers

Intervention Arm	Filter intervention 0 hours	24 hours	48 hours	72 hours	*p - over Groups*	*p – over Timepoints*
**A. Endotoxin Activity**					*0.82*	*<0.01*
**Standard of Care**	0.63 [0.55 - 0.67]	0.5 [0.42 - 0.54]	0.49 [0.38 - 0.63]	0.54 [0.43 - 0.61]		
**Oxiris**	0.62 [0.44 - 0.74]	0.59 [0.4 - 0.71]	0.4 [0.31 - 0.49]	0.55 [0.42 - 0.58]		
**Toraymyxin**	0.62 [0.57 - 0.83]	0.6 [0.29 - 0.78]	0.64 [0.36 - 0.68]	0.55 [0.44 - 0.65]		
***B.* Interleukin-6 [ng/l]**					*0.58*	*<0.001*
**Standard of Care**	2226 [945 - 6126]	786 [589 - 1612]	512 [372 - 716]	327 [115 - 567]		
**Oxiris**	3736 [1199 - 26553]	416 [342 - 2770]	317 [141 - 1309]	173 [103 - 857]		
**Toraymyxin**	2722 [509 - 6513]	1049 [218 - 2837]	316 [93 - 1838]	375 [90 - 985]		
***C.* Procalcitonin [µg/l]**					*0.16*	*<0.01*
**Standard of Care**	10.2 [2.8 - 14.4]	10.0 [3.0 - 19.5]	6.4 [2.3 - 8.2]	3.9 [1.5 - 5.1]		
**Oxiris**	12.8 [8.8 - 21]	7.9 [6.3 - 33.9]	4.7 [3.4 - 20.2]	4.4 [2.8 - 15.1]		
**Toraymyxin**	8.3 [3.3 - 107.6]	14.8 [3.1 - 49.2]	12.9 [2.6 - 24.7]	11.4 [2.8 - 28.0]		
**D. High-Mobility-Group-Protein B1 [ng/ml]**					*0.68*	*0.46*
**Standard of Care**	3.21 [2.09 - 4.03]	2.44 [2 - 4.33]	2.44 [1.5 - 2.75]	2.26 [1.92 - 3.76]		
**Oxiris**	3.19 [2.58 - 7.73]	2.41 [2.18 - 2.9]	2.37 [1.53 - 3.8]	2.82 [1.53 - 3.79]		
**Toraymyxin**	2.67 [2.14 - 3.19]	2.73 [2.44 - 4.16]	3.3 [2.39 - 4.62]	2.59 [2.04 - 4.33]		
**E. C-Reactive Protein [mg/l]**					*< 0.001*	*< 0.001*
**Standard of Care**	366 [242 - 460]	377 [317 - 455]	312 [300 - 411]	292 [242 - 344]		
**Oxiris**	182 [123 - 208]	176 [131 - 252]	164 [100 - 217]	110 [93 - 216]	*< 0.001**	

**Toraymyxin**	296 [235 - 334]	289 [239 - 375]	292 [196 - 347]	205 [87 - 337]	*0.161**	
**F. Leucocytes [10^6/l]**					*0.3*	*0.27*
**Standard of Care**	18 [13 - 22]	16 [11 - 22]	17 [10 - 20]	11 [8 - 17]		
**Oxiris**	9 [6 - 14]	9 [5 - 13]	10 [7 - 15]	10 [7 - 17]		
**Toraymyxin**	12 [7 - 19]	11 [7 - 18]	10 [7 - 19]	10 [8 - 20]		
**G. Thrombocytes [10^6/l]**					*< 0.001*	*< 0.001*
**Standard of Care**	290 [210 - 363]	220 [135 - 269]	161 [104 - 240]	138 [89 - 213]		
**Oxiris**	104 [62 - 145]	70 [41 - 94]	54 [32 - 73]	68 [33 - 72]	*0.002**	
**Toraymyxin**	167 [138 - 233]	120 [55 - 140]	64 [43 - 90]	62 [37 - 84]	*0.006**	

All parameters (median [IQR]) are given at intervention start (0 hours), 24 hours, 48 hours and 72 hours Absolute p values are given over groups and over time | * p-value for post-hoc analysis in reference to Standard of Care Arm.

Table S3: SOFA Score, Lactate levels and Vasopressor Index over time

Intervention Arm	Filter Intervention 0 hours	24 hours	48 hours	72 hours	*p - over Groups*	*p - over Times*
**A. SOFA Score**					*0.22*	*0.54*
**Standard Of Care**	12 [10 - 14]	12 [9 - 15]	12 [8 - 15]	10 [6 - 12]		
**oXiris**	14 [11 - 15]	11 [11 - 14]	14 [9 - 15]	14 [8 - 17]		
**Toraymyxin**	14 [12 - 15]	15 [14 - 17]	13 [11 - 18]	15 [11 - 18]		
**B. Lactate [mmol/l]**					*0.47*	*< 0.001*
**Standard Of Care**	2.7 [2.3 - 3.4]	1.7 [1.0 - 2.2]	1.5 [0.9 - 1.8]	0.9 [0.7 - 1.1]		
**oXiris**	3.0 [2 - 4.3]	1.6 [1.2 - 3.4]	1.2 [0.8 - 2.5]	1.2 [0.8 - 1.6]		
**Toraymyxin**	3.6 [1.4 - 5]	1.8 [1.4 - 2.1]	1.5 [1.4 - 2.1]	1.5 [1.2 - 1.6]		
**C. Vasopressor Dependency Index**					*0.95*	*< 0.001*
**Standard Of Care**	7.1 [4.6 - 8.5]	4.4 [3.0 - 5.8]	2.3 [1.0 - 2.9]	0.9 [0 – 2.0]		
**oXiris**	6.2 [4.2 - 8.3]	2.6 [0.7 - 9.2]	2.1 [0.1 - 5.5]	1.43 [0 - 6.2]		
**Toraymyxin**	5.5 [3.4 - 9.3]	4.9 [2.6 – 6.0]	2.6 [1.0 - 3.8]	1.8 [0.9 - 5.7]		

All parameters (median [IQR]) are given at intervention start (0 hours), 24 hours, 48 hours and 72 hours Absolute p values are given over groups and over time. *Further hemodynamic and ventilation parameter are reported in the Supplemental Table 3 (Supplement 2).*

Table S4: Extended Hemodynamic Parameters and PaO_2/ FiO_2 Ratio

	Filter Intervention					
Random.	0 hours	24 hours	48 hours	72 hours	*p - over Groups*	*p - over Times*
**A. PaO_2/ FiO_2 Ratio [mmHg]**					*0.43*	*0.06*
**Standard of Care**	205 [110 - 357]	192 [151 - 277]	210 [193 - 333]	231 [126 - 282]		
**Oxiris**	217 [150 - 252]	170 [133 - 258]	199 [160 - 232]	210 [171 - 244]		
**Toraymyxin**	228 [193 - 323]	247 [193 - 274]	268 [197 - 353]	236 [157 - 277]		
**B. Mean Arterial Pressure [mmHg]**					*1*	*0.03*
**Standard of Care**	68 [65 - 70]	66 [63 - 78]	69 [66 - 70]	76 [68 - 80]		
**Oxiris**	68 [60 - 72]	71 [69 - 72]	73 [71 - 85]	71 [66 - 75]		
**Toraymyxin**	72 [66 - 75]	73 [67 - 77]	72 [70 - 75]	69 [65 - 79]		
**C. Norepinephrine Dose [µg/kg/min]**					*0.75*	*< 0.001*
**Standard of Care**	0.48 [0.31- 0.57]	0.28 [0.22 - 0.32]	0.13 [0.06 - 0.2]	0.06 [0 - 0.14]		
**Oxiris**	0.42 [0.3 - 0.49]	0.17 [0.05 - 0.62]	0.12 [0.01 - 0.38]	0.09 [0 - 0.42]		
**Toraymyxin**	0.4 [0.26 - 0.61]	0.35 [0.19 - 0.41]	0.17 [0.07 - 0.27]	0.11 [0.05 - 0.35]		
**D. Inotropic Score**					*0.95*	*< 0.001*
**Standard of Care**	48 [31 - 58]	28 [22 - 37]	15 [7 - 21]	7 [0 - 15]		
**Oxiris**	42 [30 - 51]	17 [5 - 64]	14 [1 - 39]	10 [0 - 45]		
**Toraymyxin**	40 [26 - 61]	36 [20 - 41]	19 [8 - 27]	13 [6 - 35]		

All parameters (median [IQR]) are given at intervention start (0 hours), 24 hours, 48 hours and 72 hours Absolute p values are given over groups and over time.

Figure S1: Mortality at 28 Days in All patients stratified by Intervention Arm

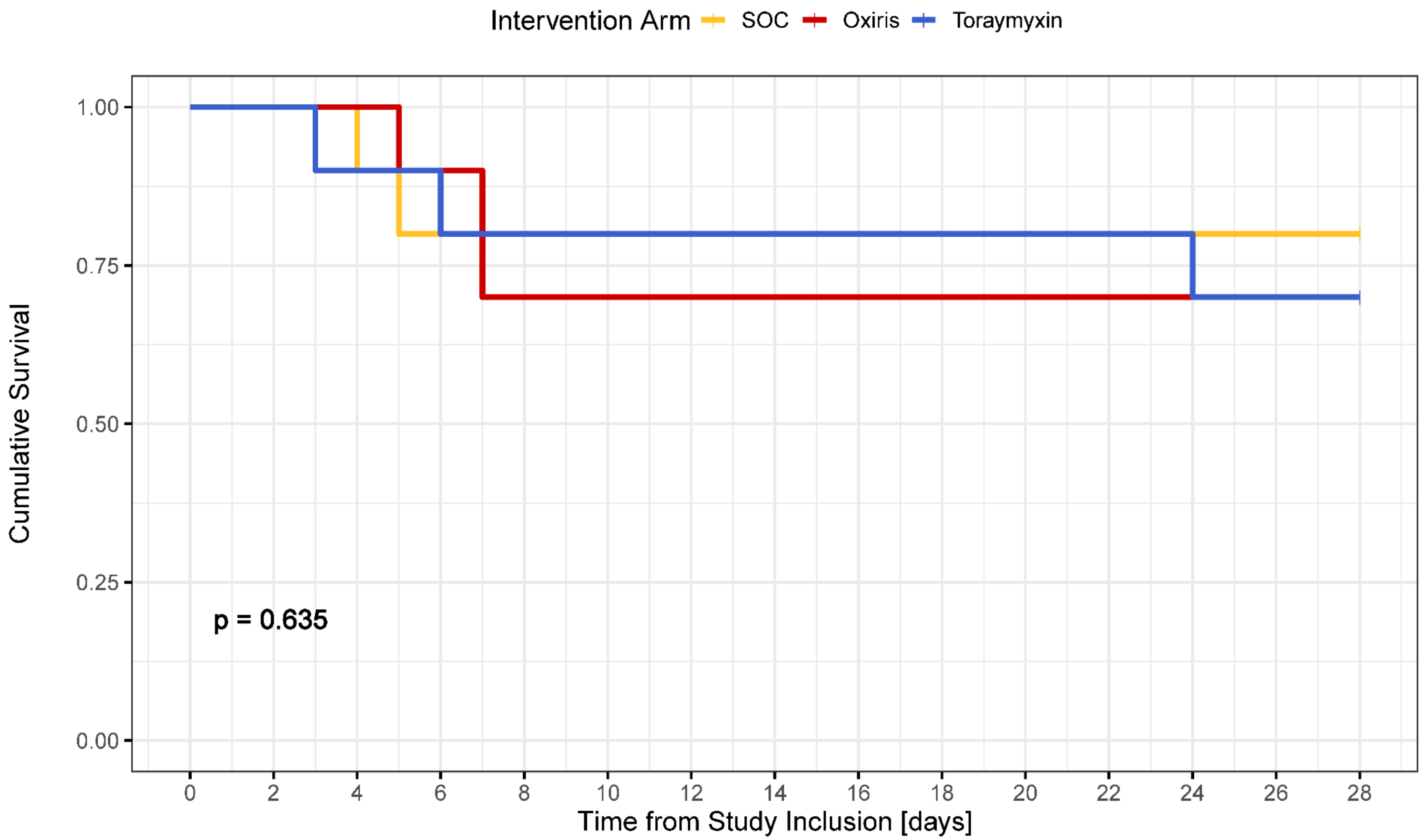

SOC	10 (0)	10 (0)	10 (0)	8 (0)	8 (0)	8 (0)	8 (0)	8 (0)	8 (0)	8 (0)	8 (0)	8 (0)	8 (0)	8 (0)	8 (8)
Oxiris	10 (0)	10 (0)	10 (0)	9 (0)	7 (0)	7 (0)	7 (0)	7 (0)	7 (0)	7 (0)	7 (0)	7 (0)	7 (0)	7 (0)	7 (7)
Toraymyxin	10 (0)	10 (0)	9 (0)	9 (0)	8 (0)	8 (0)	8 (0)	8 (0)	8 (0)	8 (0)	8 (0)	8 (0)	8 (0)	7 (0)	7 (7)

Table S5: Reported AEs & SAEs

Event	All	Standard of Care	Oxiris	Toraymyxin
**Total Reported AEs & SAEs**	14 (100%)	2 (14%)	5 (36%)	7 (43%)
**Death**	8 (57%)	2 (33%)	3 (38%)	3 (38%)
**Worsening Septic Shock**	3 (21%)	0 (0%)	0 (0%)	3 (100%)
**Filter Clotting**	2 (14%)	0 (0%)	2 (100%)	0 (0%)
**Hemodynamic Instability**	1 (7%)	0 (0%)	0 (0%)	1 (100%)

AE: Adverse Events |SAE: Serious Adverse Events | All values as number count (proportion)
