# Supplementary material for: Effects of enhanced adsorption haemofiltration versus haemoadsorption in severe, refractory septic shock with high levels of endotoxemia: the ENDoX bicentric, randomized, controlled trial
Source: Ann Intensive Care. 2023 Dec 14;13:127. doi: 10.1186/s13613-023-01224-8 (PMC10721780; doi:10.1186/s13613-023-01224-8)
Supplement: Supplementary file 2 — Additional file 2. Full Study Protocol. [file 13613_2023_1224_MOESM2_ESM.pdf]

Universität Zürich[UZH]

UniversitätsSpital Zürich

Clinical Investigation Plan

The effects of a polyethyleneimine-coated membrane (oXiris™) for hemofiltration versus polymyxin B- immobilized fibre column (Toraymyxin™) for hemoperfusion on endotoxin activity and inflammatory conditions in septic shock- A randomized controlled pilot study (ENDoX-study)

VERSION NUMBER 1.7

VERSION DATE 16.06.2016

CONFIDENTIAL

This document is confidential and the property of Medical ICU of the University Hospital Zürich and may not - in full or in part - be transmitted, reproduced, published, or otherwise used by other persons without prior written authorization from Medical ICU of the University Hospital Zürich.

Signature pages

**Study number**	*BASEC ID PB_2016-01555*
**EK-ZH-Nr.:**	2012-0458
**Swissmedic:**	2013-MD-0010
**Original Protocol Version + Date:**	Version 1.1 of 27.09.2012
**Last Protocol Version + Date**	Version 1.7 of 16.06.2016 (including Amendment 7 and 8)
**Study Title**	The effects of a polyethyleneimine-coated membrane (oXiris™) for hemo-filtration versus polymyxin B- immobilized fibre column (Toraymyxin™) for hemoperfusion on endotoxin activity and inflammatory conditions in septic shock - A randomized controlled multi-centered pilot study (ENDoX-study)

Sponsor-Investigator (Principal Investigator):

The Sponsor-Investigator has approved the investigation plan version *1.7 dated 16.06.2016*, and confirm hereby to conduct the study according to the investigation plan, current version of the World Medical Association Declaration of Helsinki, ISO 14155 norm and the local legally applicable requirements.

Sponsor-Investigator (Principal Investigator):

Marco Maggiorini
Phone +41 44 255 22 04
Fax +41 44 255 57 16

______________________________ ____
Place/Date Signature

Local Principal Investigator at study site:

I have read and understood this investigation plan and agree to conduct the trial as set out in this investigation plan, the current version of the World Medical Association Declaration of Helsinki, ISO 14155 norm and the local legally applicable requirements.

**Site**	*Stadtspital Triemli Zurich, Birmensdorferstrasse 497, CH-8063 Zürich, Switzerland*
**Principal investigator (Coordinating investigator)**	*KD Dr. med. Patricia Fodor*<br>*Phone +41 44 416 52 22*<br>*Fax +41 44 416 52 10*

______________________ ______________________

Place/Date **Signature**

Table of Contents

SIGNATURE PAGES 2

TABLE OF CONTENTS 4

1 SPONSOR INFORMATION 6

2 STUDY SYNOPSIS 7

3 STUDY FLOW CHART 10

4 LIST OF ABBREVIATIONS 12

5 BACKGROUND 13

5.1 IDENTIFICATION AND DESCRIPTION OF THE MEDICAL DEVICE TO BE INVESTIGATED 13
5.1.1 Preclinical testing 13
5.1.2 Previous clinical experience 14
5.1.3 Device risk analysis and risk assessment 14
5.1.4 Justification for the design of the clinical investigation 15

6 OBJECTIVES AND ENDPOINTS OF THE CLINICAL INVESTIGATION 16

6.1 HYPOTHESIS AND PRIMARY OBJECTIVE 16
6.2 SECONDARY OBJECTIVES 16
6.3 PRIMARY ENDPOINT 16
6.4 SECONDARY ENDPOINTS 16

7 DESIGN OF THE CLINICAL INVESTIGATION 16

7.1 STUDY DURATION 17
7.2 EXTRACORPOREAL DEVICES 17
7.2.1 The oXiris™ device 17
7.2.2 The Toraymyxin™ device 18
7.3 DEVICE ACCOUNTABILITY 18
7.4 STUDY POPULATION 18
7.4.1 Inclusion Criteria 18
7.4.2 Exclusion Criteria 19
7.5 SUBJECT RECRUITMENT 19
7.5.1 Withdrawal Criteria 19
7.6 PATIENT TREATMENT 20
7.6.1 Concomitant medication 20
7.6.2 Standard of Care group (SOC) 20
7.6.3 oXiris™ treatment group 20
7.6.4 Toraymyxin™ treatment group 21
7.6.5 Occurrence of acute renal failure during study period 22
7.7 ASSIGNMENT TO STUDY TREATMENT 22
7.8 STUDY PROCEDURES: VISITS, SAMPLING AND EVALUATED PARAMETERS 22
7.8.1 Study visits 22
7.8.2 Sampling schedule 23
7.8.2.1 Endotoxin measurements 23
7.8.2.2 Cytokine and HMGB1 measurements 23
7.8.2.3 Lactate and procalcitonin measurements 23
7.8.2.4 Sampling recommendations 23
7.8.2.5 Sampling details 23
7.8.3 Parameters and laboratory analyses 23
7.8.3.1 Laboratory analyses 23
7.8.3.2 Evaluated parameters 24
Table: SOFA score 24
7.9 PATIENTS PARAMETERS TO RECORDED IN THE CRF 25
7.9.1 Visit 1: Screening and enrolment 25

7.9.2 Visit 2: Treatment start 25
7.9.3 Visit 3 (24 hours) and Visit 4 (48 hours): Treatment period 25
7.9.4 Visit 5: Follow-up 72 hours after treatment initiation 26
7.9.5 Visit 6: Outcome 26
7.9.6 Study termination 26

8 ADVERSE EVENTS AND ADVERSE DEVICE EFFECTS 26

8.1 DEFINITIONS 26
8.2 FORESEEABLE ADVERSE EVENTS AND ADVERSE EVENTS RELATED TO CLINICAL OUTCOMES 27
8.3 RECORDING PROCEDURES 28
8.4 ASSESSMENT OF ADVERSE EVENTS 28
8.5 REPORTING OF REPORTABLE EVENTS 29
8.6 CORRECTIVE MEASURES 29
8.7 SAFETY VARIABLES 29

9 EARLY TERMINATION OR SUSPENSION OF THE INVESTIGATION 29

10 DEVIATIONS FROM THE CLINICAL INVESTIGATION PLAN 30

11 AMENDMENTS TO THE CLINICAL INVESTIGATION PLAN 30

12 REGULATORY ISSUES 30

12.1 ETHICS APPROVAL 30
12.2 REGULATORY APPROVAL 30
12.3 CONSENT 30

13 STATISTICAL CONSIDERATIONS 31

14 DATA QUALITY CONTROL AND QUALITY ASSURANCE 31

14.1 ROUTINE MONITORING 31
14.2 AUDITS AND INSPECTIONS 32

15 DATA HANDLING AND RECORD KEEPING 32

16 CONFIDENTIALITY 33

17 INSURANCE 33

18 FUNDING 33

19 STUDY REGISTRATION 34

20 PUBLICATION POLICY 34

21 REFERENCES 35

1 Sponsor Information

Sponsor-Investigator and Principal Investigator:	Prof. Dr. med. M. Maggiorini<br>Head of Medical Intensive Care Unit<br>UniversitätsSpital Zürich<br>Rämistrasse 100<br>CH- 8091 Zürich, Switzerland<br>Phone +41 44 255 22 04<br>Fax +41 44 255 57 16<br>E-mail: klinmax@usz.uzh.ch
Monitoring:	Clinical Trial Center<br>Zentrum für Klinische Forschung<br>UniversitätsSpital Zürich<br>Rämistrasse 100,<br>CH- 8091 Zürich<br>Phone +41 44 634 55 09<br>Fax: +41 44 634 55 05
Study site:	UniversitätsSpital Zürich<br>Medical Intensive Care Unit<br>Rämistrasse 100<br>CH- 8091 Zürich, Switzerland
Laboratory and other institutions involved in the trial :	Institut für Klinische Chemie (IKC)<br>UniversitätsSpital Zürich<br>Rämistrasse 100<br>CH- 8091 Zürich, Switzerland

2 Study Synopsis

**Sponsor-Investigator**	Prof. Dr. med. Marco Maggiorini
**Study Title:**	The effects of a polyethyleneimine-coated membrane (oXiris™) for hemofiltration versus polymyxin B- immobilized fibre column (Toraymyxin™) for hemoperfusion on endotoxin activity and inflammatory conditions in septic shock- A randomized controlled pilot study (ENDoX-study).
**Protocol Version and Date:**	Protocol version 1.7 of 16.06.2016
**Methodology:**	Multi centre, prospective, randomized controlled pilot study between three groups
**Study Duration:**	3 years and 6 months
**Study Centre:**	University Hospital Zürich, Rämistrasse 100, CH-8091 Zürich, Switzerland<br>Stadtspital Triemli Zurich, Birmensdorferstrasse 497, CH-8063 Zürich, Switzerland
**Investigator(s):**	KD Dr. med. Patricia Fodor
**Medical Device:**	Patients randomized in the interventional arm will be treated with Standard of Care (SOC) and hemofiltration with the oXiris™ filter.<br>The oXiris™ hemodiafilter is a heparin grafted hollow fibre membrane (AN69) which is commonly used for CRRT in patients with acute renal failure and sepsis. The surface of the AN69 membrane is treated with a positive charged polymer. This high concentration of positive charges at the membrane inner surface allows adsorbing negatively charged bacterial products such as endotoxin.
**Comparators:**	The comparators will be the following:<br>- Standard of Care,<br>- Standard of Care and hemoperfusion with Toraymyxin™ filter. The Toraymyxin™ (PMX; Toray, Tokyo, Japan) filter is a cartridge that selectively removes blood endotoxin. PMX is composed of polymyxin B covalently bonded to polystyrene-derivative fibres. It is well known that the polarity of polymyxin antibiotic binds endotoxin and has bactericidal activity.

**Rationale:**	Septic shock has a high mortality risk despite the availability of various treatments. Endotoxin, that is present in the cell walls of gram-negative bacteria, is a potent trigger of innate immunity. Endotoxin leads to an activation of a cascade with an overwhelming systemic overflow of pro- and anti- inflammatory mediators at the early phase of sepsis with generalized vascular endothelial damage, tissue injury and multi-organ failure.<br>Extracorporeal blood purification therapies aim to reduce the circulating level of endotoxin. Different extracorporeal blood purification systems are available. The oXiris™ device comprises a surface treated AN69 membrane capable to adsorb a large spectrum of plasma cytokines, such as IL-6 and HMGB1 protein. The positively charged inner surface of the membrane allows absorbing negatively charged bacterial products such as endotoxin. From an historical perspective, filters containing AN69-based membranes have been the most commonly used products for CRRT in the management of critically ill patients and a substantial volume of published data exist.<br>Another extracorporeal endotoxin removal therapy is the hemoperfusion with ToraymyxinTM (PMX) filter, which is a cartridge selectively removing blood endotoxin. PMX is composed of polymyxin B covalently bonded to polystyrene-derivative fibres. It is well known that the polarity of the polymyxin B antibiotic binds endotoxin and has bactericidal activity. Therefore, the rationale underlying extracorporeal therapy with PMX is to remove circulating endotoxin by adsorption.
**Objectives:**	<u>Primary objective:</u> evaluate the endotoxin activity and removal with two extracorporeal blood purification treatments in septic shock patients.<br><u>Secondary objectives:</u> evaluate the effects of the two blood purification treatments on cytokine and HMGB-1 removal, haemodynamics, safety, and outcome.
**Endpoints:**	Primary endpoint:<br>Measurement of the endotoxin activity 72 hours after treatment initiation.<br>Secondary endpoints:<br>1. Measurement of IL-6 and HMGB1 at T0 i.e. patient inclusion, at T24 hours, T48 hours, and T72 hours after treatment initiation<br>2. Routine measurements of norepinephrine dose, inotropic score, and vasopressor dependency index at T0, T24, T48, T72<br>3. Routine measurements of volume requirement at T0, T24, T48, T72<br>4. Routine measurements of PO2/FiO2, MAP at T0, T24, T48, T72<br>5. Lactate and procalcitonin measurements at T0, T24, T48, T72<br>6. SOFA score calculation at T0, T24, T48, T72<br>7. Incidence of renal failure, ICU length of stay, patient survival at D28<br>8. Recording of AEs/SAEs
**Number of Subjects:**	30 patients in total

**Diagnosis and Inclusion Criteria:**	• Patients with septic shock, defined as: persistent catecholamine dependency with a vasopressor index > 3 after fluid resuscitation with at least 30ml/kg/KG prior to time of randomisation, and at least ONE of the following criteria: metabolic acidosis, neurologic dysfunction (GCS ≤ 14), renal dysfunction (RIFLE Classification: staging Injury or Failure) or hepatic dysfunction (Transaminase ≥ twice upper limit of normal).<br>• Male and Female patients ≥18 years<br>• Endotoxin levels ≥0.6 IU EAA within 24h from the time of suspected septic shock. If the first Endotoxin level is ≥ 0.4 ≤ 0.6 IU EAA, the measurement should be repeated 2 -3 times within the screening period of 24 hours..
**Exclusion Criteria:**	• Contraindications on ethical grounds<br>• Endotoxin levels <0.6 IU EAA<br>• Pregnancy or breast feeding<br>• Neutropenia (circulating neutrophils <500/µl)<br>• Immune-suppressive therapy or<br>Steroid medication ≥30mg/d Prednisone Equivalent<br>• Use of Vasopressin (Pitressin)<br>• Organ transplantation within the last 12 months<br>• Terminally ill patients classified as “do not resuscitate”<br>• Have active bleeding or are at increased risk for bleeding, defined as platelets count after substitution >30 G/L; INR >4; and at least Substitution of more than 2 erythrocyte concentrate within 6 hours<br>• History of sensitivity to polymyxin B or to anticoagulant (heparin) HIT or allergy to heparin<br>• Need for extracorporeal membrane oxygenation (ECMO)
**Study Schedule:**	First patient In: 01/08/2013<br>Last patient Out: 01/02/2017
**Statistical Methodology:**	The effect of oXiris™ on endotoxin activity is not well described in the literature, which makes the sample size calculation difficult. Therefore, this trial was designed as a pilot study analysing 30 patients in order to collect data enabling an accurate design of future investigations.
**Statement/ Regulatory:**	This study will be conducted in compliance with the CIP, the current version of the Declaration of Helsinki, the European Directive on medical devices 93/42/EEC, the EN-ISO Norm 14155, EN-ISO 14971 as well as all national legal and regulatory requirements.

3 Study Flow Chart

STUDY FLOW CHART

Study Periods	***Screening & Enrolment***	***Treatment start***	***oXiris™ Treatment period 48hrs (2x24h)***	***Toraymyxin™ and/or SOC Treatment period (2x2h and/or SOC)***	***Follow up 72hrs***	***Outcome D28 ± 7days***
Study visits	***V1***	***V2***	***V3+ V4***	***V3+ V4***	***V5***	***V6***
Procedures						
Demographics	*x*					
Medical History	*x*					
Infection site, type of infection, documented microbial agents	*x*					
Antibiotic therapy	*x*					
In- /Exclusion Criteria	*x*					
Pregnancy test	*x*					
Catheter implementation for extracorporeal circulation		*x*				
EAA measurements	*(x)*	*x*	*x*	*x*	*x*	
IL-6 and HMGB1 measurements		*x*	*x*	*x*	*x*	
Lactate and procalcitonin measurements, pH	*x*	*x*	*x*	*x*	*x*	
Informed consent/Consent of relatives					*x*	
Hemodynamic parameters	*x*	*x*	*x*	*x*	*x*	
Ventilation parameters		*x*	*x*	*x*	*x*	
Catecholamine doses	*x*	*x*	*x*	*x*	*x*	
Volume requirement		*x*	*x*	*x*	*x*	
Urine output (ml/d)		*x*	*x*	*x*	*x*	
SOFA score		*x*	*x*	*x*	*x*	
SAPS II Score	*x*	*x*				
Concomitant therapy	*x*	*x*	*x*	*x*	*x*	*x*
AE/SAE	*x*	*x*	*x*	*x*	*x*	*x*
Patient outcome					*x*	*x*
Study Termination Form						*x*

Flow chart: Study visits (V1-V6) for oXiris™,Toraymyxin™ and Standard therapy groups in septic shock

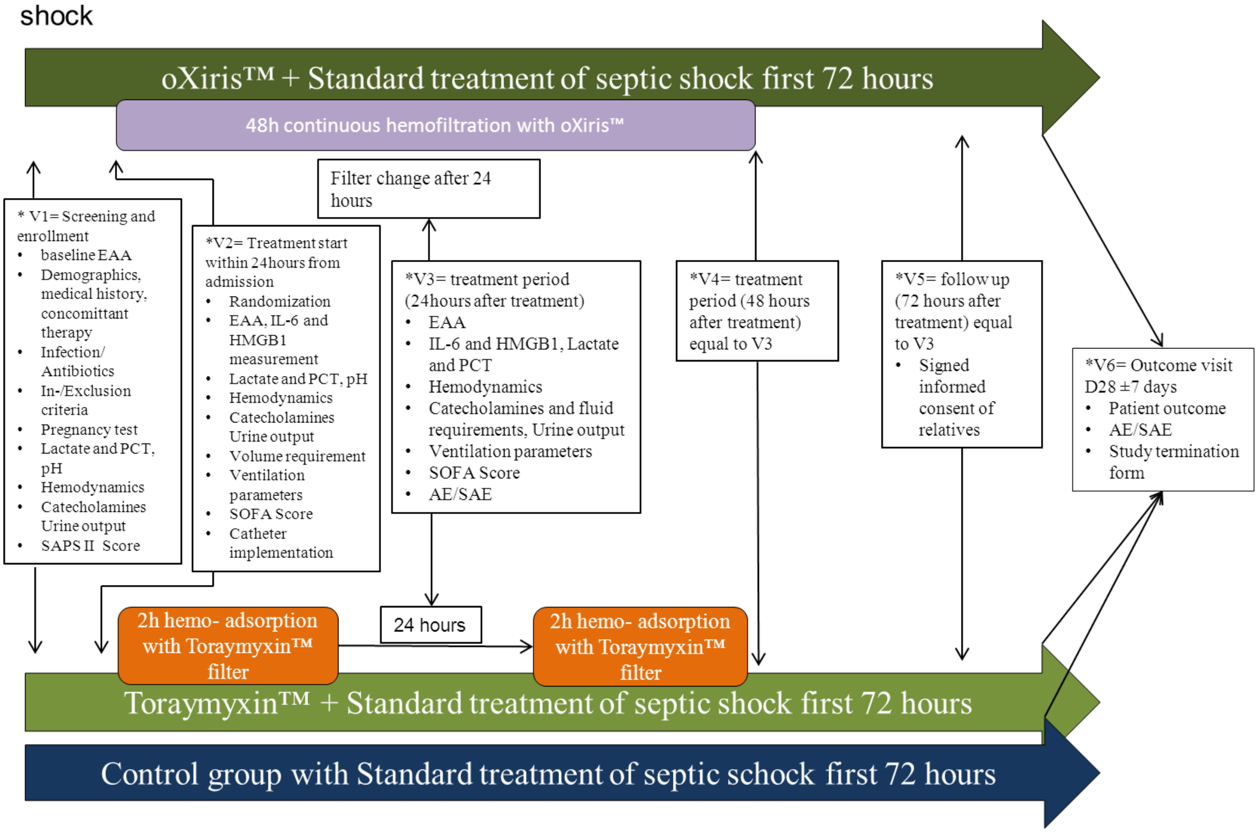

* For details see also Study flow chart above

4 List of Abbreviations

ACCP/SSCM	American College of Chest Physicians/Society of Critical Care Medicine
AE	Adverse Event
AKI	Acute kidney injury
CIP	Clinical Investigation Plan
CO	Cardiac output
CVP	Central venous pressure
CRF	Case Report Form
CRRT	Continuous Renal Replacement Therapy
CVVH	Continuous veno- venous hemofiltration
CVVHD	Continuous veno- venous haemodialysis
CVVHDF	Continuous veno- venous haemodiafiltration
EAA	Endotoxin Activity Assay
ELWI	Extra-vascular Lung Water Index
GCP	Good Clinical Practices
HMGB1	High-Mobility Group protein B1
HR	Heart Rate
ICU	Intensive Care Unit
IEC	Independent Ethics Committee
IL-6	Interleukin 6
ISO	International Standardization Organisation
LPS	Lipopolysaccharide
MAP	Mean arterial pressure
PTT	Partial Thrombin Time
PVPI	Pulmonary Vascular Permeability Index
SOC	Standard of Care
SOFA score	Sequential Organ Failure Assessment score
TNF α	Tumor Necrosis Factor α

5 Background

5.1 Identification and description of the medical device to be investigated

Septic shock has a high mortality risk despite the availability of various treatments (1). The systemic sepsis response starts with the recognition of an invading organism or its toxin. Bacterial-associated toxins are some of the principal components of gram-negative (endotoxin) and gram-positive organisms (lipotechoic acid) (2-4).
Endotoxin, a complex lipopolysaccharide (LPS) that is present in the cell walls of gram-negative bacteria, is a potent trigger of innate immunity. The activated immune cells, principally monocytes/macrophages are able to recognize and destroy the pathogenic agents and the endotoxins by phagocytosis. These cells also release a series of mediators (cytokines) that activate other cells. Tumor necrosis factor-alpha (TNF-α), Interleukin (IL-1 and IL-6) and high-mobility group box protein 1 (HMGB1), a 30 kDa alarmin, are important pro-inflammatory cytokines which activate complements and coagulation factors.
This activation of the immune cascade can lead to an overwhelming systemic overflow of pro- and anti-inflammatory mediators at the early phase of sepsis with generalized endothelial damage, tissue injury and multi-organ failure; the leading cause of death in septic patients.

High flow Haemofilter Device for Blood Purification
Extracorporeal blood purification therapies have been proposed for sepsis as adjuvant treatment. A recent advance in blood purification is oXiris™ (Gambro Industries, Meyzeiu, France), an extracorporeal filter capable of removing endotoxin and cytokines. The oXiris™ device contains an AN69-based membrane, which adsorbs a large spectrum of plasma cytokines, such as IL-6, IL-8, IL-10, and TNFα, and has the potential to reduce cytokine plasma levels (5-9). Furthermore, it has been recently demonstrated that HMGB1 can be significantly removed using a "surface-treated" version of AN69 (AN69ST) in vitro (10). The surface treatment of AN69 to produce AN69ST involves the application of a positively-charged polymer (polyethyleneimine: PEI) at relatively low concentration to the inner (blood-contacting) surface of the negatively charged AN69 membrane. Moreover, the high concentration of positive charges at the membrane inner surface allows negatively-charged bacterial products, such as endotoxin, to be adsorbed. On the other hand, this manufacturing approach has no effect on the AN69-related cytokine-adsorbing characteristics, which have been demonstrated to be preserved with oXiris™.
From an historical perspective, filters containing AN69-based membranes have been commonly used products for CRRT in the management of critically ill patients. For example, continuous veno-venous hemofiltration using the AN69 membrane has been reported to be effective in improving immune dysfunction and increasing monocyte HLA-DR expression (11). This combination of characteristics for oXiris™ potentially represents a major advantage over isolated hemoperfusion, especially regarding organ failure management during septic shock. This device is intended to be a complete blood purification option covering several aspects of the sepsis condition, including renal support for acute renal failure, a clinical entity frequently observed in septic shock patients (12).Preliminary investigations and justification of the study

5.1.1 Preclinical testing

Promising in vitro and animal data have already been reported with oXiris™. In an in vitro study, bovine plasma contaminated with endotoxin from an E. coli source was circulated in a closed circuit in contact with oXiris™ at a flow rate of 250 ml/min. Sixty six per cent (66%) of the endotoxin present at the beginning of the experiment was adsorbed after a one hour treatment (13). Moreover, the use of oXiris™ during a 6-hour hemofiltration session in septic

pigs significantly improved haemodynamics as compared to pigs treated with a standard hemofiltration membrane. Interestingly, pigs that were treated with the oXiris™ membrane had serum endotoxin levels lower than pigs from the control group (13).

5.1.2 Previous clinical experience

Recently, an observational uncontrolled study enrolling 16 patients with severe sepsis or septic shock has shown the use of oXiris™ is associated with a significant decrease in inflammatory markers, such as procalcitonin (PCT) and interleukin 6 (IL-6). In addition, treatment with oXiris™ is associated with a significant improvement of several clinical parameters; including mean arterial pressure, norepinephrine requirements, and SOFA score (14, 15).
Hemofiltration with the oXiris™ filter is commonly performed in patients with acute renal failure and severe sepsis or septic shock, requiring CRRT.

5.1.3 Device risk analysis and risk assessment

The medical literature does not provide definitive information regarding the percentage of septic shock patients who develop severe acute renal failure and require renal replacement therapy. However, according to several publications based on critically ill populations with sepsis or severe sepsis, a reasonable estimate is that about 50% of patients with severe sepsis eventually require renal replacement therapy (16, 17). In the interventional group, a certain percentage of patients who do not develop acute renal failure severe enough to require renal replacement therapy will, nevertheless, receive extracorporeal therapy and be exposed to associated risks. However, morbidity specifically related to renal replacement therapy has drastically decreased over time due to improvement in machines, circuits and membranes.

In a study published by Payen et al. in 2009, the application of 'standard' continuous hemofiltration (conventional dose and filter) to a population with severe sepsis, (irrespective of the presence of renal failure), resulted in a significantly prolonged requirement for organ support (longer time to wean from mechanical ventilation and catecholamines) (17). However, the risk/benefit assessment for this study may not have been well aligned as the study assessed only a "renal support dose" rather than the potential benefits of a "sepsis modulation" blood purification therapy such as high-volume hemofiltration or high adsorption hemofiltration, which is the technique to be evaluated in the current study during a limited time period i.e. 48 hours.

In addition, all the risks related to renal replacement therapy should be viewed in the context of the potential benefits of the oXiris™ set. The oXiris™ set enables the removal of endotoxin and cytokines, compounds which are integral in the development of sepsis-related multiple organ failure.

The kinetics of endotoxin removal with extracorporeal blood purification therapy is not well described in the literature. A recent review showed conflicting results on endotoxin levels in patients treated with Polymyxin B-immobilized filters.
The rationale of the extracorporeal therapy with PMX is to remove circulating endotoxin by adsorption, thus preventing progression of the biological cascade of sepsis. Overall, nine randomized controlled trials (mainly published in Japan) investigated the PMX on outcomes (blood pressure, the use of vasoactive drugs mortality and endotoxin levels) (18-24). Endotoxin concentrations decreased after PMX treatment in most of the studies, but did not significantly change in the European randomized controlled trial (25). A recent review reported similar adverse events, including clotting of the device when compared to CRRT

(16). Especially no adverse events indicative of nephrotoxicity and neurotoxicity were reported in a recent review (16).

The following is a list of problems that could be theoretically encountered with the use of extracorporeal blood purification treatments:
• Circuit and filter thrombosis, possibly leading to blood loss;
• Heparin-associated bleeding or heparin-induced thrombocytopenia;
• Hemodynamic instability, mainly due to hypovolemia;
• Various catheter-related complications, including inadvertent arterial puncture, local bleeding, hematoma, pneumothorax, haemothorax, retroperitoneal bleeding or cardiac arrhythmia, thrombosis, infection, mechanical problems (e.g. malpositioning);
▪ Electrolyte disturbances;
▪ Undesired losses of water-soluble compounds, including drugs and trace elements.

5.1.4 Justification for the design of the clinical investigation

Little is known about the endotoxin activity using extracorporeal blood purification. In a recent observational study of 16 patients with severe sepsis or septic shock treated with oXiris™, the authors showed a significant decrease in inflammatory markers (PCT and IL-6) and a significant improve in clinical parameters (MAP, norepinephrine requirements and SOFA score) (28). However, it is poorly described whether the hemodynamic improvement was correlated to the endotoxin activity or other unknown factors.
Similarly, the Toraymyxin™ capacity to lower blood endotoxin levels and immunologic substances (IL-6) is not well understood, although the reported favourable effects on haemodynamics.
This pilot study aims to evaluate these two different blood purification treatments in terms of endotoxin activity and cytokines removal.
Patients will be followed-up during three days to collect parameters such as catecholamine support, hemodynamic parameters, SOFA score, lactate and procalcitonin which are parameters usually collected in septic shock. The survival rate at day 28 will be collected as well.

6 Objectives and endpoints of the clinical investigation

6.1 Hypothesis and primary objective

The primary objective of the study is to evaluate the endotoxin activity and removal with two extracorporeal blood purification treatments in septic shock patients.

6.2 Secondary objectives

The secondary objectives of the study comprise the effects of the two blood purification treatments on cytokine and HMGB-1 removal, haemodynamics, safety, and outcome.

6.3 Primary endpoint

The study primary endpoint is the measurement of the endotoxin activity 72 hours after treatment initiation.

6.4 Secondary endpoints

The secondary endpoints are the following:

- Measurement of IL-6 and HMGB1 T0 i.e. patient inclusion, at T24 hours, T48 hours, and T 72 hours after treatment initiation;
- Routine measurements of norepinephrine dose, inotropic score, and vasopressor dependency index T0, T24, T48 and T72;
- Haemodynamics: MAP, CVP, HR, CO, ELWI, PVPI T0, T24, T48 and T72
- Routine measurements of volume requirement T0, T24, T48 and T72;
- Routine measurements of ventilation parameters PO2/FiO2, SvO2 T0, T24, T48 and T72;
- Urine output (ml/d)
- Lactate, procalcitonin and pH measurements at T0, T24, T48 and T72 after treatment initiation;
- SOFA score calculation at T0, T24, T48 and T72 ;
- Incidence of renal failure, ICU length of stay
- Patient survival at D28;
- Recording of AEs/SAEs

7 Design of the clinical investigation

The clinical study design is the following: Pilot,
Proof of concept,
Prospective, Open,
Multi-centre,
Randomized,
Controlled trial between three parallel groups

The study is open as the standard of care of septic shock treatment does not include extracorporeal support.

7.1 Study duration

The study is planned to start 01/08/2013 (First patient in) and stop in 01/02/2017 (Last patient out).The patient enrolment is expected to last 3 years and 6 month.

7.2 Extracorporeal devices

7.2.1 The oXiris™ device

The product name is oXiris™ Set, which consists of the following:
• a heparin grafted hollow fibre haemofilter/dialyzer
• a tubing circuit
• a cartridge plate
• an effluent bag (5 Litres)
The hemodiafilter is comprised of a hollow fibre membrane with an effective surface area of 1.5m².
This hollow fibre membrane is composed of:
• a co- polymer of Acrylonitrile and sodium methallyl sulfonate (AN69)
• a surface treatment agent: Polyethyleneimine
• Heparin that is grafted to the membrane
The oXiris™ Set is sterilized by Ethylene Oxide.
The oXiris™ Set is intended for use only with the PRISMAFLEX Control Unit equipped with software version 4.00 or later in providing continuous fluid management and renal replacement therapies.

oXiris™ indications and contraindication
The oXiris™ set is intended for use in the following veno-venous therapies: CVVH, CVVHD, CVVHDF. This product is CE marked for the intended use described below and manufactured according to the essential requirements described in the directive 2007/47/EEC and it belongs to Class III device according to this directive.
The system (oXiris™ and Prismaflex) is intended for patients who have acute renal failure, fluid overload or both.
The oXiris™ set is currently not intended for the use in patients with septic shock without acute renal failure.
All information related to manufacturing of study product is available in the Investigator's brochure.
oXiris™ is contra-indicated for patients who have a known allergy to heparin or Heparin Induced Thrombocytopenia (HIT).

oXiris™ labelling
Each investigational device will bear the following information:
• Identification label bearing all mandatory and regulatory information, including sterilization and shelf life;
• Barcode label for product manufacturing traceability purposes.
A label stating "for investigational purpose only" will be affixed to each box.
The solutions used to perform CRRT will be Gambro Bicarbonate fluids such as Hemosol B0, Prismasol 2, Prismasol 4, and Phoxilium.

7.2.2 The Toraymyxin™ device

Toraymyxin™ (PMX; Toray, Tokyo, Japan) is a cartridge selectively removing blood endotoxin. It consists of the following:

- A polycarbonate case containing a fibre composite tissue based on polystyrene,
- immobilized polymyxin B trough a covalent binding,
- a tubing circuit.

The blood flows radially and homogenously from a central pipe with holes in its sidewall to the composite fibre fabric wound around it.

It is well known that polymyxin-B antibiotic binds endotoxin and has bactericidal activity. Polymyxin-B has a strong affinity to endotoxin and is able to bind the lipid A portion of endotoxin through ionic and hydrophobic interactions. Intravenous injection of polymyxin-B has significant nephrotoxic and neurotoxic effects. However, covalently immobilized polymyxin-B on the adsorbents of PMX does not leak out into the blood stream, thus allowing the clinical application without the known toxic effects of polymyxin-B.

Toraymyxin™ indications and contraindications

Toraymyxin™ is intended for endotoxin removal in patients with severe sepsis and septic shock. Toraymyxin™ has been CE-marked according to ECC 93/42 for medical devices since 1998. It has been used in Japan for over 15 years with more than 70,000 patients treated so far.

The parenteral application of polymyxin B is limited by its toxicity.

7.3 Device accountability

The study products intended for the clinical study must be stored in a safe place with restricted access and used only to treat the patients included in the study under the sole supervision of the investigator. The principal investigator (or delegated person) is responsible for recording both the product supplied as well as the number of product units used during the study, in addition to material.

The principal investigator is solely responsible for keeping records of updated documents according to the following requirements:

a) The date of receipt;
b) Identification of each investigational device (batch number);
c) The expiry date;
d) The date or dates of use;
e) Subject identification;
f) Date on which the investigational device was returned/withdrawn from subject, if applicable;
g) The date of return of unused, expired or malfunctioning investigational devices, if applicable.

At each visit, the Study Monitor must ensure that products have been duly counted, and that the products are being used according to their specifications.

7.4 Study population

7.4.1 Inclusion Criteria

The following inclusion criteria should be fulfilled at the time of enrollment within 24h of suspected septic shock.

Patients with septic shock, defined as: persistent catecholamine dependency with a vasopressor index > 3 after fluid resuscitation with at least 30ml/kg of i.v. fluid administered

prior to time of randomization, and at least ONE of the following criteria: metabolic acidosis, neurologic dysfunction (GCS ≤ 14), renal dysfunction, (RIFLE Class Injury or Failure), or hepatic dysfunction (Transaminases ≥ twice upper limit of normal).

- Male and Female patients ≥18 years
- Endotoxin levels ≥ 0.6 IU within 24h from the time of suspected septic shock. If the first Endotoxin level is ≥ 0.4 ≤ 0.6 IU EAA, the measurement should be repeated 2-3 times within the screening period of 24 hours.

7.4.2 Exclusion Criteria

The presence of any one of the following exclusion criteria will lead to exclusion of the subject:
Contraindications on ethical grounds
Endotoxin levels <0.6 IU EAA Pregnancy
or breast feeding
Neutropenia (circulating neutrophils <500/µl)
Immune-suppressive therapy or Steroid medication ≥30mg/d Prednisone Equivalent
Use of Vasopressin (Pitressin)
Organ transplantation within the last 12 months Terminally
ill patients classified as "do not resuscitate" Have active
bleeding or are at increased risk for bleeding

- Have a platelets count after substitution >30 G/L
- Have an INR >4
- Substitution of more than 2 erythrocyte concentrate within 6 hours

History of sensitivity to polymyxin B or to anticoagulant (heparin) HIT or allergy to heparin
Need for extracorporeal membrane oxygenation (ECMO)

7.5 Subject Recruitment

Patients who met all inclusion criteria and none of the exclusion criteria (see above) will be considered for enrolment. Informed Consent will be obtained as described in section 12.3.

7.5.1 Withdrawal Criteria

Patients are free to withdraw from the study at any time without having to justify their decision. When a patient decides to withdraw from a study, he/she will be systematically contacted and given the opportunity to provide information about the reason(s) of his/her withdrawal and possible occurrence of an adverse event.

If at any time the investigator considers that malfunctions/AEs could possibly have led to a deficiency in the patient's treatment, the treatment in question shall be discontinued and replaced by a standard treatment.
Patients must be withdrawn from the study if any of the following occurs:

- Any event which, in the opinion of the investigator, may either endanger the health of the patient, or may adversely influence the result of the study;
- Patients refusing or not considered capable, as judged by the investigator, of following instructions of the study;
- In case of clotting of three oXiris™ circuits within 24 hours, or a filter downtime for more than 6 hours, the treatment will be stopped.
- The need for extracorporeal membrane oxygenation (ECMO)
- Patients who die within 72 hours from admission

Withdrawn patients after randomization will be replaced. The reason for withdrawal should be recorded in the Case Report Form (CRF).
If withdrawal is related to the possible occurrence of adverse events, the patient will be followed up according to the adverse event procedure.

7.6 Patient treatment

Irrespective of their randomization, all patients will receive Standard of Care treatment for sepsis, according to the Surviving Sepsis Guidelines (27), including volume resuscitation, administration of vasopressors, antibiotic therapy, hemodynamic monitoring, nutrition, organ support e.g. mechanical ventilation, corrective measures for metabolic abnormalities, and renal replacement therapy, when appropriate.

7.6.1 Concomitant medication

Any medication or treatment required for the welfare and health of the patients enrolled in the study will be administered at the investigator's discretion. All such concomitant medications shall be reported on the CRF during the patient's stay in ICU i.e. from the signature of the discharge from ICU.
Vasopressin and corticosteroids for the treatment of septic shock are not allowed in the study. In patients without pre-existing chronic corticosteroid treatment, corticosteroids are not allowed in the study.

7.6.2 Standard of Care group (SOC)

Patients randomized into the SOC group will receive Standard of Care treatment for sepsis, according to the Surviving Sepsis Guidelines (see above) (27).

7.6.3 oXiris™ treatment group

Vascular access
In the oXiris™ group, patients will receive extracorporeal therapy over a venous double-lumen catheter, inserted through the jugular, subclavian or femoral vein. The catheter will be inserted by a trained physician according the guidelines for performing ultrasound guided vascular cannulation (28).

Priming of the circuit
Although the oXiris™ membrane is pre-heparinised, it possesses residual capacity for the adsorption of heparin.
Therefore, in order to saturate the membrane, the oXiris™ set is to be rinsed with a 2-Liter priming solution containing 5000 IU/L of unfractionated heparin.
For patients with bleeding risk: once priming is complete, the set blood circuit still contains heparinised solution. Depending on an individual patient's bleeding risk, the physician must decide if additional priming using 500 ml of a non-heparinised solution is necessary.

Anticoagulation strategy
Heparin will be used as an anticoagulant for the extracorporeal circuit except for high bleeding risk patients, for whom no anticoagulant will be used. The heparin dose is adjusted to achieve an anti-Xa Level of 0.3-0.5 according to our CRRT SOP.

Study treatment duration
The oXiris™ treatment will be applied during 48 hours of treatment i.e. two (2) oXiris™ sets each one used for 24 hours.

oXiris™ set change

The oXiris™ set will be changed every 24 hours. The filter change should be done within a 30-45 min time frame.

Treatment parameters

Mode: CVVHDF: 20% diffusion-80% convection
Dose: 35 ml/h/kg*
Reinfusion: 50% Predilution, 50% Postdilution
Blood flow rate (Qb): 150 ml/min at least.
Filtration Fraction should be inferior to 15%.
Once hemodynamic stabilization is achieved, fluid overload should be managed according to the patient clinical needs.
*prescribed dose to secure a delivered dose of min 25ml/h/kg

Cessation of study treatment with oXiris™

The use of oXiris™ set cannot be extended beyond the 48 hours treatment period.
If a patient requires renal replacement therapy beyond the 48 hours study treatment period, he/she will be treated with the usual CRRT set (see below).

7.6.4 Toraymyxin™ treatment group

Vascular access

In the Toraymyxin™ group, patients will receive extracorporeal therapy over a venous double-lumen catheter, inserted through the jugular, subclavian or femoral vein. The catheter will be inserted by a trained physician according the guidelines for performing ultrasound guided vascular cannulation (28).

Priming of the circuit

The Toraymyxin™ filter is to be rinsed with a 4-liter priming solution followed by 500 ml of heparinised saline solution (4U/ml).

Anticoagulation strategy

Heparin 8IU/kg will be administered as maintenance. The maximum maintenance dose allowed for any patient is 2000U/hr. In patients with a high risk for bleeding, no anticoagulant will be used. The heparin dose is adjusted to achieve an ACT of 150 to 180s and an anti-Xa Level of 0.3-0.5 according to our CRRT SOP.

Study treatment duration

Patients randomized to the Toraymyxin™ group will be treated for 2 sessions lasting 2 hours each, with an interval of 24 hours after the treatment initiation between the 2 sessions, according to the EUPHAS trial (16).

Cessation of study treatment with Toraymyxin™

The use of Toraymyxin™ cannot extend beyond the 24 hour treatment period.
If CRRT is needed, the hemofiltration is performed with the MultiFiltrate™ (Fresenius) and AV1000S haemofilter or the Prismaflex®-system and the Prismaflex ST150 haemofilter according to our SOP. During the treatment period with the Toraymyxin™ filter the CRRT is stopped.

Treatment parameters Mode:
Direct hemoperfusion Inlet
pressure: <250mmHg
Maximum pressure: 500 mmHg
Blood flow rate: 150 ml/min

7.6.5 Occurrence of acute renal failure during study period

Initiation of Renal Replacement Therapy

Patients with acute renal failure requiring renal replacement therapy will be treated with continuous renal replacement therapy irrespective of their randomization group. Criteria to start CRRT are the following:

- Metabolic acidosis with a pH<7.2
- HCO3- <16mmol/l
- Urine- output <20ml/h
- Uncontrolled hyperkalaemia
- Fluid overload

The oXiris™ product cannot be used in the Standard of Care group nor and Toraymyxin™ treatment group. The hemofiltration in these groups will be performed with the MultiFiltrate™ (Fresenius) and the AV1000S haemofilter or the Prismaflex®-system and the Prismaflex ST150 haemofilter according to our SOP.

Patient medical care after clinical study closure

After the clinical investigation has been completed, the usual medical care will be given.

7.7 Assignment to study treatment

Randomization

All eligible patients will be randomly assign to either conventional SOC treatment according to the Surviving Sepsis Campaign Guidelines (27), or SOC treatment with oXiris™ hemofiltration, or SOC treatment with Toraymyxin™ hemoperfusion within 24 hours of admission to the ICU. Patients will be distributed into these 3 groups, in accordance with the randomization table kept in the investigator's office. The randomization list will be performed using a computer generated (simple random number) scheme.
The allocation sequence is concealed in sealed envelopes held at the study centre, which is available 24 hours a day.
According to paragraph 7.5.1 withdrawn patients will be replaced in order to analyse 10 patients per group.

Treatment allocation

Treatment allocation will be done according to the usual delivery process. After randomization, the investigator is responsible for completing the randomization list and the accountability documents to record reception of study product.

7.8 Study Procedures: visits, sampling and evaluated parameters

7.8.1 Study visits

There will be 6 study visits:

- V1: Screening and enrolment visit
- V2: Treatment start
- V3 and V4: Treatment period
- V5: Follow up visit 72 hours after treatment initiation
- V6: Outcome visit at Day 28 ±7 days after randomization

7.8.2 Sampling schedule

7.8.2.1 Endotoxin measurements

Endotoxin activity (EAA) will routinely be measured for patients admitted to the ICU if the diagnosis of Septic Shock is considered. Is the EAA level above the cut off threshold value 0.6 an evaluation for the study inclusion will be done (the result can be used for screening and enrolment (V1)). Furthermore the EAA will be measured at treatment start (V2), 24 hours after treatment initiation (V3), 48 hours after treatment initiation (V4), and 72 hours (V5) after treatment initiation (see treatment schedule p. 8). Blood samples will be obtained through an arterial line.

7.8.2.2 Cytokine and HMGB1 measurements

IL-6 and HMGB1 will be measured at treatment start (V2), 24 hours after treatment initiation (V3), 48 hours after treatment initiation (V4), and 72 hours (V5) after treatment initiation (see treatment schedule p. 8). Blood samples will be obtained through an arterial, peripheral or central venous line. IL-6 and HMGB1 will be also measured in the effluent line in order to get information about the adsorption by the oXiris™ filter.

7.8.2.3 Lactate and procalcitonin measurements

Lactate and procalcitonin will be measured at patient enrolment (V1), 24 hours after treatment initiation (V3), 48 hours after treatment initiation (V4), and 72 hours (V5) after treatment initiation (see treatment schedule p. 8). Blood samples will be obtained through an arterial, peripheral or central venous line.

7.8.2.4 Sampling recommendations

Sampling, handling and storage details will be described in more detail in a study specific procedure and presented during the study training.

7.8.2.5 Sampling details

Blood samples: No additional puncture of the patient is necessary.
In total ~70 ml blood during the entire study will be collected for study purposes.
All samples will be destroyed after the study.

7.8.3 Parameters and laboratory analyses

7.8.3.1 Laboratory analyses

All laboratory tests will be performed at the Institute of Clinical Chemistry (University Hospital Zürich) according to standard procedures.

Cytokines (IL-6) measurements will be performed with the Luminex technique at the Institute of Clinical Chemistry (University Hospital Zürich).
HMGB1 measurements will be performed by an enzyme-linked immunoabsorbant assay method.

Endotoxin Activity will be measured in EDTA whole blood using an in vitro diagnostic test (EAA™, Spectral Diagnostics Inc., Ontario, Canada). Whole blood samples will be collected from an arterial vascular access in tubes containing EDTA as anticoagulant and immediately brought to the lab at room temperature and analysed. Prior to analysis blood is thoroughly mixed by gentle inversion for 30 seconds. The test principle is the following: Specific anti-endotoxin antibodies bind to endotoxin (or lipopolysaccharide, a major cell wall constituent of

gram negative bacteria) in the blood specimen thereby forming an endotoxin-antibody complex. The opsonisation of this immune complex by complement factors primes neutrophils in the blood sample to enhance their respiratory burst in response to zymosan.

The oxidants released by the neutrophils react with luminol contained in the reaction mixture and emit chemiluminescence that can be measured using a luminometer.
The measurement is done using 3 tubes: a negative control without addition of the specific anti-endotoxin antibody, the tube where the endotoxin activity in the patient's sample is measured and a positive control after maximum stimulation of respiratory burst by an addition of an excess of exogenous endotoxin. The result of the test is given as the ratio of the endotoxin activity in the patient's sample measured by chemoluminescence and the maximum chemiluminescence of the positive control, after correction of both measurements for the basal luminescence reflecting the non-specific oxidative burst of the patient's neutrophils. Measurement results are accepted if the coefficient of variation is less than 30% for results below 0.2, and less than 15% for results equal or higher than 0.2. Otherwise the analysis is repeated. Results less than 0.4 indicate a low endotoxin activity level, those between 0.4 and 0.59 an intermediate, and those equal or higher than 0.6 a high endotoxin activity level.

7.8.3.2 **Evaluated parameters**

- Inotropic score: (dobutamine dose x1) + (adrenaline dose x 100) + (noradrenalin x 100), where all doses are expressed as µg/kg/min
- Vasopressor dependency index: Inotropic score / MAP (mmHg) x10
- Volume requirements
- Ventilation parameters
- Urine output (ml/d)
- SOFA score calculation
- SAPS score
- EAA measurement
- IL-6 and HMGB1 measurements
- Lactate, CRP and procalcitonin measurements, pH

Table: SOFA score

**SOFA score**	**0**	**1**	**2**	**3**	**4**
**Respiration**<br>PaO2/FiO2 mmHg	>400	≤400	≤300	≤200	≤100
**Coagulation**<br>Platelets x106/L	>150	≤150	≤100	≤50	≤20
**Liver**<br>Bilirubin (µmol/l)	<20	20-32	33-100	101-203	>203
**Cardiovascular**<br>Hypotension (mmHg)	None	MAP<70	Dop or any ≤5	Dop >5 or Norepi≤0.1, Epi ≤0.1	Dop >15, Norepi>0.1, Epi >0.1
**CNS**<br>Glasgow coma scale	15	13-14	10-12	6-9	<6
**Renal**<br>Creatinin (µmol/l)	<106	106-168	169-300	301-433	>434

Dopamine (Dop), epinephrine (Epi), norepinephrine (Norepi) doses in µg/kg/min

7.9 Patients parameters to recorded in the CRF

7.9.1 Visit 1: Screening and enrolment

- Pregnancy test
- Patients date of birth, gender, height and weight
- Medical history and type of admission
- Concomitant therapy
- Infection site, type of infection and documented microbial agents
- Antibiotic therapy
- Inclusion and exclusion criteria
- Baseline blood sample: PCT, Lactate, pH
- Endotoxin activity (EAA) measurement
- SAPS score
- Hemodynamic parameters
- Catecholamine doses

7.9.2 Visit 2: Treatment start

- Randomization to either SOC treatment, or SOC with oXiris™ hemofiltration, or SOC with Toraymyxin™ hemoperfusion
- EAA measurement
- IL-6 and HMGB1 measurement
- Catheter implementation
- Hemodynamic parameters: MAP, CVP, HR, CO, ELWI, PVPI
- Catecholamine doses: Epinephrine, norepinephrine, dobutamine dose
- Volume requirement
- Ventilation parameters: SaO2, FiO2, SvO2
- Urine output (ml/d)
- SOFA score, SAPS II ScorePCT, Lactate, pH

7.9.3 Visit 3 (24 hours) and Visit 4 (48 hours): Treatment period

- EAA measurement
- IL-6 and HMGB1
- PCT, Lactate, pH
- SOFA score
- Catecholamine doses: Epinephrine, norepinephrine, dobutamine dose
- Volume requirement
- Hemodynamic parameters: MAP, CVP, HR, CO, ELWI, PVPI
- Ventilation parameters: SaO2, FiO2, SvO2
- Urine output (ml/d)
- AE/SAE
- In the SOC with oXiris™ treatment and SOC with Toraymyxin™ treatment groups:
    - Date and time of initiation and termination of each treatment, anticoagulation usage
    - Documentation of achievement of the treatment period or reason for premature disconnection.
- In the SOC treatment group:
    - If renal replacement therapy has to be performed: type of RRT, type of membrane, treatment conditions, duration, and anticoagulation.

7.9.4 Visit 5: Follow-up 72 hours after treatment initiation

- EAA measurement
- Signed and dated informed consent of relatives and independent doctor (see p. 30).
- IL-6 and HMGB1
- PCT, Lactate, pH
- SOFA score
- Catecholamine doses: Epinephrine, norepinephrine, dobutamine dose
- Hemodynamic parameters: MAP, CVP, HR, CO, ELWI, PVPI
- Ventilation: SaO2, FiO2, SvO2
- Urine output (ml/d)
- AE/SAE
- Informed consent

7.9.5 Visit 6: Outcome

- Discharge from ICU
- Discharge from hospital
- Survival 28 days ± 7 days after randomization. Patients will be contacted by phone, if the patient is no longer in hospital. In case, documented attempts to contact the patient have failed, a rapid contact with the patient's general practitioner will be initiated. To be in line with the data protection requirements the patients gives the consent to contact the general practitioner in the informed consent form. Date of death (if applicable)
- AE/SAE

7.9.6 Study termination

When a patient completes the study, the study termination form has to be completed.

8 Adverse events and adverse device effects

8.1 Definitions

Adverse Device Effect: Adverse event related to the use of an investigational medical device. This includes any AE resulting from insufficiencies or inadequacies in the instructions for use, the deployment, the implantation, the installation, the operation, or any malfunction of the investigational medical device. This includes any event that is a result of a use error or intentional misuse.

Adverse Events: Any untoward medical occurrence, unintended disease or injury or any untoward clinical signs (including an abnormal laboratory finding) in subjects, users or other persons whether or not related to the investigational medical device. This includes events related to the investigational device or the comparator and to the procedures involved (any procedure in the clinical investigational plan. For users or other persons this is restricted to events related to the investigational medical device.

Device Deficiency: Inadequacy of a medical device with respect to its identity, quality, durability, reliability, safety or performance, including malfunctions, use errors, and inadequate labelling.

Serious Adverse Device Effect: Adverse device effect that has resulted in any of the consequences characteristic of a serious adverse event.

Serious Adverse Event: Adverse event that

- led to a death,
- led to a serious deterioration in health of subject, that either resulted in
  1) a life-threatening illness or injury, or
  2) a permanent impairment of a body structure or a body function, or
  3) in-patient or prolonged hospitalisation, or
  4) medical or surgical intervention to prevent life-threatening illness or injury or permanent impairment to a body structure or a body function,
- led to foetal distress, foetal death or a congenital abnormality or birth defect.

This includes device deficiencies that might have led to a SAE if

- suitable action had not been taken or
- intervention had not been made or
- if circumstances had been less fortunate.

These are handled under the SAE reporting system. A planned hospitalization for pre-existing condition, or a procedure required by the Clinical Investigation Plan, without serious deterioration in health, is not considered to be an SAE.

A **Serious Deterioration** in state of health includes:

a) life-threatening illness,
b) permanent impairment of a body function or permanent damage to a body structure,
c) a condition necessitating medical or surgical intervention to prevent a) or b) (e.g. clinically relevant increase in the duration of a surgical procedure or a condition that requires hospitalisation or significant prolongation of existing hospitalisation)
d) any indirect harm as a consequence of an incorrect diagnostic or in vitro diagnostic test results when used within manufacturer's instructions for use,
e) foetal distress, foetal death or any congenital abnormality or birth defects.

Unanticipated Serious Adverse Device Effect: Serious adverse device effect that by its nature, incidence, severity or outcome has not been identified in the current version of the risk analyses report.
If there is any doubt about whether or not an AE has to be considered serious, the Principle Investigator should be contacted.

8.2 Foreseeable Adverse Events and Adverse Events related to Clinical Outcomes

All Adverse Events (AE) will be collected during study days 0 to 28 (or until death).

The following events will be considered as clinical outcomes:

- Death related to septic shock, that is, related to septic shock or a sequel of sepsis based on the interpretation of the investigator
- Cardiovascular events: the need for vasoactive drugs or hypotension.
- Respiratory events: decreased PaO2/FiO2 mechanical ventilation, hypoxia, acute respiratory distress syndrome, acute lung injury or respiratory failure
- Hepatic events: hepatic injury or liver dysfunction that leads to an increase from baseline in the serum level of bilirubin
- Hematologic/coagulation events: coagulopathy, disseminated intravascular coagulation, thrombocytopenia or thrombocytosis
- Systemic inflammatory response related criteria: tachypnoea, hypopnoea, leucocytosis, leucopenia, hypothermia, hyperthermia, tachycardia and bradycardia

8.3 Recording Procedures

Clinical investigators and ultimately the Principal Investigator (PI) have the primary responsibility for AE identification and documentation,

All adverse events and adverse device effects should be documented. Depending on the nature of the event the reporting procedures below should be followed. Any questions concerning adverse event reporting should be directed to the Principal Investigator in the first instance.

The investigator is responsible for reporting all (S)AEs occurring during the course of the study. AEs (serious or non-serious) observed by the investigator and/or reported by the subject must be recorded in the patient file and subsequently in the CRF, i.e. the period of time from the first (= signature of informed consent and/or the presumed will of the participant) to the last protocol-specific procedure (Visit 6 on Day 28 or date of death) regardless of the study product relation assessment.

All observed or volunteered adverse device events (serious or non-serious) and abnormal test findings, regardless of treatment group or suspected causal relationship to the investigational device or study treatment(s) will be recorded.

AEs or abnormal test findings felt to be associated with the study treatment(s) will be followed until the event (or its sequelae) or the abnormal test finding resolves or stabilizes at a level acceptable to the investigator.

All AEs, serious and non-serious, will be fully documented in the appropriate CRF. For each AE, the investigator will provide the onset, duration, intensity, treatment required, outcome and action taken with the investigational product.

For all AEs, sufficient information will be pursued and/or obtained so as to permit an adequate determination of the outcome of the event (i.e., whether the event should be classified as a SAE) and an assessment of the casual relationship between the AE and the investigational study treatment.

8.4 Assessment of Adverse Events

After collecting information about an occurred event, the investigator assesses if it is a reportable event and has to be reported to the IEC and competent authority, respectively.

One of the following reporting criteria must be fulfilled:

- an SAE that could have been caused by an IMD or occurred in connection with a procedure carried out during the clinical trial.
- any investigational medical device deficiency that might have led to a SAE if
  - o suitable action had not been taken or
  - o intervention had not been made or
  - o if circumstances had been less fortunate,
- new findings/updates in relation to already reported events.

8.5 Reporting of Reportable Events

The Sponsor-Investigator is responsible for reporting to the IEC and competent authority according to the following details.

- Provision of reportable events to the IEC and competent authority according to the following time frames: SAE which indicates an imminent risk of death, serious injury, or serious illness and that requires prompt remedial action for other patients/subjects, users or other persons or a new finding to it: **immediately**, but not later than **2 calendar days** after awareness by Sponsor-Investigator of a new reportable event or of new information in relation with an already reported event.
- Any other reportable event related to the medical device under investigation or due to study related procedures: **immediately**, but not later than **7 calendar days** following the date of awareness by the Sponsor-Investigator of the new reportable event or of new information in relation with an already reported event.
- Send a list of all reportable events and a safety report of the trial subjects on a yearly basis to the IEC and competent authority over the entire duration of the clinical trial.

The form ”Clinical investigations of medical devices: Notification of serious adverse events concerning Switzerland” by Swissmedic is to be used.

8.6 Corrective Measures

Corrective measures considered as important for the protection of the health and safety of the study subjects can and should of course be taken immediately by the sponsor and the investigators.

8.7 Safety variables

According to the current local regulation:
• An “Annual Safety Report”/”Development Safety Update Report”, containing all safety issues linked to the study product (including control product) must be sent each year to EC and Competent Authorities.

9 Early termination or suspension of the investigation

The study will be discontinued if the investigators judge it necessary for any reason. Possible scenarios are:

- >50% SAE possible related to the study device
- Technical issues with the study device

If the investigation is terminated prematurely or suspended, the IEC and the competent

authority will be informed promptly and provided with the reason(s) for the termination or suspension by the sponsor-investigator.

10 Deviations from the clinical investigation plan

The Investigator is not allowed to deviate from the CIP, unless in emergency circumstances, where deviations from the CIP protect the rights, safety and well-being of the subjects. Such deviations may precede without prior approval of the competent authority and the IEC, but will be documented and reported to the sponsor and the regulatory authorities as soon as possible.

11 Amendments to the clinical investigation plan

Deviations should be reviewed to determine the need to amend the CIP or to terminate the investigation.
The IEC and competent authority must be informed about any amendment to the CIP.
Any change or addition to this protocol requires a written protocol amendment that must be approved by the investigator before implementation. Amendments affecting the safety of subjects, the scope of the investigation or the scientific quality of the study, require additional approval of the IEC.
Examples of amendments requiring such approval are:
A significant change in study design (e.g. deletion of a control group)
An increase in number of invasive procedures to which subjects are exposed
Addition or deletion of a test procedure for safety monitoring

12 Regulatory issues

This study will be conducted in compliance with the CIP, the current version of the Declaration of Helsinki, the European Directive on medical devices 93/42/EEC, the EN-ISO Norm 14155, EN-ISO 14971 as well as all national legal and regulatory requirements (29, 30).

12.1 Ethics approval

The Sponsor-Investigator will obtain approval from the Independent Ethics Committee before starting the clinical investigation.

12.2 Regulatory Approval

The Sponsor-Investigator will obtain approval from competent authority before starting the clinical investigation.

12.3 Consent

The current study involves a population of vulnerable patients. Patients with septic shock may need emergency interventions including sedation for intubation before the study participation can be discussed. Patients with septic shock often present with confusion and are not judicious. In these situations, in which the patient is not capable to decide to participate or not, the relatives are asked about the presumptive will (advance directive). In all patients, an independent doctor assures the medical treatment with his signature. In all patients, written informed consent from the patient himself will be obtained, once the patient is judicious. This will usually take several days to weeks. The right of the patient to refuse to participate will be respected without giving reasons. Data obtained will be destroyed, except data for security

reasons, which have to be stored by law (EN ISO 14155). Because the data obtained can also influence the security of other patients treated with the medical device within studies or not, their interest of security is rated above the right of the individual patient involved in the study. After finishing the study these data are not stored in the study records but in the patient's medical history record. In the study record a key to the medical history record will be deposited.

If the patient remains not judicious, the consent of a previous nominated legal representative by the patient gives consent according to 'Kantonalem Patientengesetz § 29 Abs. 4 and the Erwachsenenschutzrecht ZGB Art 377 ff' (31).

In a minority of cases, the patient will be conscious and capable to decide to participate or not. In these cases, consent to enter the study will be sought from each subject after a full explanation has been given, an information leaflet offered and time allowed for consideration. Signed informed consent will be obtained. The right of the patient to refuse to participate will be respected without giving reasons. With the signature of an independent doctor, the medical treatment is assured.

After the subject has entered the study the clinician remains free to give alternative treatment to that specified in the CIP at any stage if he/she feels it is in the subject's best interest, but the reasons for doing so should be recorded. In these cases, the subject remains within the study for the purposes of follow-up and data analysis. All subjects are free to withdraw at any time from the CIP treatment without giving reasons and without prejudicing further treatment.

13 Statistical considerations

As mentioned, the endotoxin activity is not well described in the literature, which makes a sample calculation difficult. Therefore, this trial was designed as a pilot study analysing 30 patients in order to collect data enabling an accurate design of future investigations. Dependent and independent continuous variables will be compared using Wilcoxon and Mann-Whitney, respectively. In cases of serial measures repeated measures statistics will be applied. For multiple comparisons overall tests will be used: ANOVA for continuous variables and Kruskal-Wallis tests for ordinal scaled variables. Categorical data will be compared using Fisher's exact and $\chi 2$ test. The normal distribution of study variables will be confirmed using Kolmogorov-Smirnov test. A P value < 0.05 will be considered statistically significant. To obtain adjusted differences in outcomes for the two study populations, logistic regression will be performed to control for factors or confounders that are significantly different ($p < 0.05$) between compared groups. Data will be analysed using the Statistical Package for Social Sciences (SPSS Windows, version 19.0; SPSS Inc., Chicago, IL).

14 Data Quality Control and Quality Assurance

14.1 Routine Monitoring

Regular monitoring visits at the investigator's site prior to the start and during the course of the study will help to follow up the progress of the clinical investigation, to assure utmost accuracy of the data and to detect possible errors at an early time point.

CRFs, progress notes and copies of laboratory and medical test results will be available for monitoring. The monitor will review all or a part of the CRFs and written informed consents.

The study will be monitored regularly by an independent monitor according to a pre-defined

monitoring plan.

The monitor will verify that compliance with the CIP is maintained. Any deviation from the CIP will be discussed with the clinical investigators and documented as well as reported to the sponsor.

During monitoring visits, completeness and correctness of the data in the case report forms as well as consistency with the source data will be verified and include following information:

- Patient's informed consent obtained prior to the start of the study, if applicable. Exceptions see section 6.3
- Number of patients enrolled, included, withdrawn or completed
- Verification of source data
- Compliance with inclusion and exclusion criteria
- Compliance with sampling procedures
- Potential deviation from protocol
- Safety issues (i.e. potential observed AEs, SAEs)

14.2 Audits and Inspections

A quality assurance audit/inspection of this study may be conducted by the competent authority or IEC, respectively. The quality assurance auditor/inspector will have access to all medical records, the investigator's study related files and correspondence, and the informed consent documentation that is relevant to this clinical study.

The investigator will allow the persons being responsible for the audit or the inspection to have access to the source data/documents and to answer any questions arising. All involved parties will keep the patient data strictly confidential.

15 Data Handling and Record Keeping

The investigators will use paper case report forms (CRF), one for each enrolled study participant, to be filled in with all relevant data pertaining to the subject during the study. All subjects who either entered the study or were considered not-eligible or were eligible but not enrolled into the study additionally have to be documented on a Screening log. The investigator will document the participation of each study subject on the Enrolment Log.

All information in the paper CRF will be completed in a neat legible manner with a BLACK or a BLUE BALL POINT pen to ensure clarity of reproduced copies in the CRF. All corrections in the paper CRF will be made in a way to not obscure the original entry. The correct data will be inserted, dated and
initialled by the investigator. Data that are not available or not done will be made clear by adding NA or ND. CRFs will be kept current to reflect subject status at each phase during the course of study. Subjects are not to be identified in the CRF by name. Appropriate coded identification (Subject Number) will be used.

It will be assured that any authorised person, who may perform data entries and changes in the CRF, can be identified. A list with signatures and initials of all authorised persons will be filed in the study site file and the trial master file, respectively. The defined Dataset will contain all data of the study, and is only accessible by defined persons involved in the study and secured by a password. Changes in the Dataset will be marked with the initials of the designed investigator staff. A declaration ensuring accuracy of data recorded in the case

report form will be signed by the investigator.

Documented medical histories and narrative statements relative to the subject's progress during the study will be maintained. These records will also include the following: originals or copies of laboratory and other medical test results (e.g. ECGs, EEG, x-ray reports) which must be kept on file with the individual subject's CRF. If source data is available on a print out (e.g. laboratory values, x-ray reports), this print out is to be kept in a source data folder, and the data necessary for the study is to be transferred to the CRF.

Essential documents must be retained for at least 10 years after the regular end or a premature termination of the respective study (VKlin Art. 25). Any patient files and source data must be archived for the longest possible period of time according to the feasibility of the investigational site, e.g. hospital, institution or private practice.

16 Confidentiality

Data generation, transmission, archiving and analysis of personal data within this study, strictly follows the current Swiss legal requirements for data protection. Prerequisite is the voluntary approval of the subject given by signing the informed consent prior start of participation of the clinical trial.

Individual subject medical information obtained as a result of this study is considered confidential and disclosure to third parties is prohibited. Subject confidentiality will be further ensured by utilising subject identification code numbers to correspond to treatment data in the computer files.

Such medical information may be given to the subject's personal physician or to other appropriate medical personnel responsible for the subject's welfare, if the patient has given his/her written consent to do so.

Data generated as a result of this study are to be available for inspection on request by the monitors, by the IEC and the competent authorities.

17 Insurance

Insurance is covered by “Haftpflichtversicherung für den Kanton Zürich betreffend das UniversitätsSpital Zürich“ (Policy no: 14.970.888).

Any damage developed during the course of the study is covered by this insurance. So as not to forfeit their insurance cover, the subjects themselves must strictly follow the instructions of the study personal. Subjects must not be involved in any other medical treatment without permission of the principal investigator (emergency excluded). Medical emergency treatment must be reported immediately to the investigator. The investigator must also be informed instantly, in the event of health problems or other damages during or after the course of study treatment.
The investigator will allow delegates of the insurance company to have access to the source data/documents as necessary to clarify a case of damage related to study participation. All involved parties will keep the patient data strictly confidential.
A copy of the insurance certificate will be placed in the Investigator’s Site File.

18 Funding

Gambro Company is funding this study with an unrestricted grant of 150’000 CHF.

19 Study Registration

The study is/will be registered in the local trial registry of the University Hospital Zürich („Studienregister USZ“). The study will also be registered at trials.gov.

20 Publication policy

After the statistical analysis of this trial the sponsor will make every endeavour to publish the data in a medical journal.

21 References

1. Angus DC, Linde-Zwirble WT, Lidicker J, et al. Epidemiology of severe sepsis in the United States: analysis of incidence, outcome, and associated costs of care. Crit Care Med 2001;29(7):1303-1310.
2. Hattar K, Grandel U, Moeller A, et al. Lipoteichoic acid (LTA) from Staphylococcus aureus stimulates human neutrophil cytokine release by a CD14-dependent, Toll-like-receptor-independent mechanism: Autocrine role of tumor necrosis factor-[alpha] in mediating LTA-induced interleukin-8 generation. Crit Care Med 2006;34(3):835-841.
3. Manocha S, Feinstein D, Kumar A. Novel therapies for sepsis: antiendotoxin therapies. Expert Opin Investig Drugs 2002;11(12):1795-1812.
4. Marshall JC, Foster D, Vincent JL, et al. Diagnostic and prognostic implications of endotoxemia in critical illness: results of the MEDIC study. J Infect Dis 2004;190(3):527-534.
5. Bouman CS, van Olden RW, Stoutenbeek CP. Cytokine filtration and adsorption during pre- and postdilution hemofiltration in four different membranes. Blood Purif 1998;16(5):261-268.
6. De Vriese AS, Colardyn FA, Philippe JJ, et al. Cytokine removal during continuous hemofiltration in septic patients. J Am Soc Nephrol 1999;10(4):846-853.
7. Haase M, Silvester W, Uchino S, et al. A pilot study of high-adsorption hemofiltration in human septic shock. Int J Artif Organs 2007;30(2):108-117.
8. Peng Z, Pai P, Han-Min W, et al. Evaluation of the effects of pulse high-volume hemofiltration in patients with severe sepsis: a preliminary study. Int J Artif Organs 2010;33(8):505-511.
9. Strobel WM, Gurke T, Schifferli JA. Lowering plasma levels of complement factor D with AN69 dialysis membranes. J Clin Apher 1999;14(4):188-189.
10. Yumoto M, Nishida O, Moriyama K, et al. In vitro evaluation of high mobility group box 1 protein removal with various membranes for continuous hemofiltration. Ther Apher Dial 2011;15(4):385-393.
11. Peng Z, Pai P, Hong-Bao L, et al. The impacts of continuous veno-venous hemofiltration on plasma cytokines and monocyte human leukocyte antigen-DR expression in septic patients. Cytokine 2010;50(2):186-191.
12. Uchino S, Kellum JA, Bellomo R, et al. Acute renal failure in critically ill patients: a multinational, multicenter study. JAMA 2005;294(7):813-818.
13. Rimmele T, Assadi A, Cattenoz M, et al. High-volume haemofiltration with a new haemofiltration membrane having enhanced adsorption properties in septic pigs. Nephrol Dial Transplant 2009;24(2):421-427.
14. Caravetta P LA, Severi L, et al. Clinical evaluation of new CRRT membrane with endotoxin and cytokines removal properties. SMART congress 2011.
15. Candidi F CM, Caravetta P et al. . Continous renal replacement therapy with an adosrbent membrane in postooperative septic cardiac patients: A clinical experience. . EACTA/Abstracts/Free oral sessions 211 2012.
16. Cruz DN, Antonelli M, Fumagalli R, et al. Early use of polymyxin B hemoperfusion in abdominal septic shock: the EUPHAS randomized controlled trial. JAMA 2009;301(23):2445-2452.
17. Payen D, Mateo J, Cavaillon JM, et al. Impact of continuous venovenous hemofiltration on organ failure during the early phase of severe sepsis: a randomized controlled trial. Crit Care Med 2009;37(3):803-810.

18. Nakamura T, Ushiyama C, Suzuki S, et al. Effect of polymyxin B-immobilized fibre hemoperfusion on sepsis-induced rhabdomyolysis with acute renal failure. Nephron 2000;86(2):210.
19. Nakamura T, Ushiyama C, Suzuki Y, et al. Hemoperfusion with polymyxin B-immobilized fibre in septic patients with methicillin-resistant Staphylococcus aureus-associated glomerulonephritis. Nephron Clin Pract 2003;94(2):c33-39.
20. Nakamura T, Ushiyama C, Suzuki Y, et al. Hemoperfusion with polymyxin B immobilized fibres for urinary albumin excretion in septic patients with trauma. ASAIO J 2002;48(3):244-248.
21. Nemoto H, Nakamoto H, Okada H, et al. Newly developed immobilized polymyxin B fibres improve the survival of patients with sepsis. Blood Purif 2001;19(4):361-368; discussion 368-369.
22. Suzuki H, Nemoto H, Nakamoto H, et al. Continuous hemodiafiltration with polymyxin-B immobilized fibre is effective in patients with sepsis syndrome and acute renal failure. Ther Apher 2002;6(3):234-240.
23. Tani T, Hanasawa K, Endo Y, et al. Therapeutic apheresis for septic patients with organ dysfunction: hemoperfusion using a polymyxin B immobilized column. Artif Organs 1998;22(12):1038-1044.
24. Uriu K, Osajima A, Hiroshige K, et al. Endotoxin removal by direct hemoperfusion with an adsorbent column using polymyxin B-immobilized fibre ameliorates systemic circulatory disturbance in patients with septic shock. Am J Kidney Dis 2002;39(5):937-947.
25. Vincent JL, Laterre PF, Cohen J, et al. A pilot-controlled study of a polymyxin B-immobilized hemoperfusion cartridge in patients with severe sepsis secondary to intra-abdominal infection. Shock 2005;23(5):400-405.
26. Wernovsky G, Wypij D, Jonas RA, et al. Postoperative course and hemodynamic profile after the arterial switch operation in neonates and infants. A comparison of low-flow cardiopulmonary bypass and circulatory arrest. Circulation 1995;92(8):2226-2235.
27. Dellinger RP, Levy MM, Rhodes A, et al. Surviving sepsis campaign: international guidelines for management of severe sepsis and septic shock: 2012. Crit Care Med 2013 Feb;41(2):580-63728.
28. Troianos CA, Hartman GS, Glas KE, et al. Guidelines for performing ultrasound guided vascular cannulation: recommendations of the American Society of Echocardiography and the Society of Cardiovascular Anesthesiologists. J Am Soc Echocardiogr 2011;24(12):1291-1318.
29. International Conference on Harmonisation of Technical Requirements for Registration of Pharmaceuticals for Human Use (ICH) adopts Consolidated Guideline on Good Clinical Practice in the Conduct of Clinical Trials on Medicinal Products for Human Use. Int Dig Health Legis 1997;48(2):231-234.
30. HMG, Heilmittelgesetz (Bundesgesetz über Heilmittel und Medizinprodukte, RS 812.21).
31. VKlin, Verordnung über klinische Versuche mit Heilmitteln (RS812.214.2)
